# Impacts of alcohol health warning labels in a real-world setting: protocol for a randomised controlled trial among supermarket customers in Barcelona

**DOI:** 10.1136/bmjopen-2025-103464

**Published:** 2026-01-21

**Authors:** Daša Kokole, Maria Neufeld, Daniela Correia, Jürgen Rehm, Carina Ferreira-Borges, Erin Hobin, Pol Rovira, Lidia Segura Garcia, Joan Colom Farran

**Affiliations:** 1 World Health Organization Regional Office for Europe, Copenhagen, Capital Region of Denmark, Denmark; 2 Maastricht University Care and Public Health Research Institute, Maastricht, LI, Netherlands; 3 Centre for Addiction and Mental Health, Toronto, Ontario, Canada; 4 University of Toronto Department of Psychiatry, Toronto, Ontario, Canada; 5 University of Toronto Dalla Lana School of Public Health, Toronto, Ontario, Canada; 6 University Medical Center Hamburg-Eppendorf Department of Psychiatry and Psychotherapy, Hamburg, HH, Germany; 7 Public Health Ontario, Toronto, Ontario, Canada; 8 University of Victoria Canadian Institute for Substance Use Research, Victoria, British Columbia, Canada; 9 Public Health Agency of Catalonia, Barcelona, Spain

**Keywords:** Knowledge, Health policy, PUBLIC HEALTH

## Abstract

**Introduction:**

Alcohol is causally related to more than 200 diseases and injuries. Alcohol health warning labels are a promising intervention to address alcohol-related harm with multiple possible roles, but research on its real-world impacts is lacking. This study aims to experimentally evaluate the impact of exposure to two types of content (responsibility and cancer message) and positioning of the message (front or back) on knowledge of alcohol causing cancer as the primary outcome and alcohol consumption behaviour, intentions, risk perception, emotional response, product appeal and policy support as secondary outcomes. The study also aims to assess the potential testing effect of pre-measurement on the primary outcome.

**Methods and analysis:**

Participants (of the legal drinking age in Spain (18 years or older), purchased at least one alcoholic beverage (with alcohol by volume (ABV) ≥ 1.2% for their own consumption and speaking Catalan or Spanish) will be recruited outside of supermarkets in Barcelona after purchasing alcohol, randomly assigned into one of the eight experimental groups, complete a baseline questionnaire (with half of the sample answering baseline questions measuring knowledge) and receive label stickers displaying either responsibility or cancer message, and applied to either front or back of every alcohol container they have purchased. They will complete follow-up surveys measuring the primary and secondary outcomes 1 week and 1 month after the intervention, either online or via telephone. The key hypotheses are that the label containing a cancer message will have a greater impact on the primary and secondary outcomes compared with the responsibility label. To evaluate the impact of health warning labels on knowledge of alcohol causing cancer, logistic regression will be employed to model the probability of a correct response as a function of the key independent variables, with results reported as ORs. Secondary outcomes will be modelled through linear regressions for continuous variables, and through logistic regressions for dichotomic variables or categorical variables that will be dichotomised a priori. The target sample size is 1300 participants.

**Ethics and dissemination:**

The study has been approved by the Ethical Committee for Research with Medicines (CEIm) IDIAP Jordi Gol (24/228-P) and the Ethical Research Committee of the WHO (ERC.0004213). The results will be disseminated in peer-reviewed journals, on social media and policy fora in national, European and global context, and will inform WHO and European Union-level policy recommendations.

**Funding:**

European Commission, Directorate General for Health and Food Safety, SANTE/2022/SI2.883729.

**Trial registration number:**

NCT06915298. https://clinicaltrials.gov/study/NCT06915298

**STRENGTHS AND LIMITATIONS OF THIS STUDY**

- The experiment presents a methodology for evaluating the understudied real-world impacts of alcohol health warning labels in a naturalistic setting without necessity of retailer involvement or existing legislation, recruiting the participants directly after the purchase of alcoholic beverages and affixing labels before consumption.
- The study investigates the impacts of both the label message content and positioning of the label on the container.
- Exposure to the label will depend on the amount of alcohol purchased at the time of the recruitment and participants’ consumption timing, which means it may differ among participants.
- Participants recruited in front of the supermarkets that agree to participate in the study may differ systematically from the general population in ways that affect their response.

## Introduction

 Alcohol is a health-harming substance that is causally related to more than 200 diseases and injuries.^1^ In 2019, almost 240 000 people in the European Union (EU) died from alcohol-caused health conditions, with death fractions attributable to alcohol highest among young people.^2^ Alcohol causes at least seven types of cancer, including colon and female breast cancers, the most common ones in the EU.^3^ To mitigate the health consequences, efforts should be made to decrease alcohol consumption among the general population. The European Framework for Action on Alcohol 2022–2025^4^ focuses on six areas, including the ‘best buys’ of the WHO (pricing increases via taxation or other means, marketing bans, availability restrictions), as well as health service responses, providing health information and community action. Health information provision has a focus on labelling of alcoholic beverage containers, which enables wide reach at the point of sale, pour and consumption. Recent evidence shows the necessity of informing the public about the relationship between alcohol and cancer, due to low awareness, including in Spain.^5 6^ At present, Spain has no legal requirements to display health warning labels (HWLs) on alcohol containers^7^; however, certain alcohol producers display pictograms related to pregnancy, drinking and driving and age restrictions or messages on responsible drinking as part of their voluntary commitments.^8 9^

Current data on labelling effectiveness on knowledge and behavioural outcomes from real-world settings mainly come from tobacco and nutrition fields.^10 11^ In the alcohol field, there is scarce evidence on the impact the alcohol HWLs displayed in a real-world setting have over longer term, as many of the existing mandatory warnings have not been evaluated or are poorly designed,^12^ and voluntary warnings often reflect the alcohol producers’ aim to be perceived as socially responsible, rather than provide visible and evidence-based health information.^13^ Most scientific studies of enhanced alcohol HWLs are conducted in an artificial online or lab-based setting with short duration of exposure and short follow-up periods, often immediately after the exposure.^14 15^ These kinds of studies often focus on behavioural precursors, such as risk perception and intentions, with mixed results that likely result from the methodology heterogeneity.^16^,^22^

To date, only one study has evaluated the longer-term impacts of alcohol labels in a real-world setting: the Yukon study, in which the monopoly store in Whitehorse, the capital city of the territory, included health information through stickers on almost all products, but this study had to be interrupted due to industry interference which could have also impacted the results.^23^ This study found that including a cancer warning in combination with low-risk drinking guidelines and information on standard drinks led to an increase in knowledge of the relationship between alcohol and cancer and increased support for alcohol policies,^24^ as well as a long-term decrease in per capita ethanol sold relative to a comparison site without the intervention labels.^25^ The Yukon study also indicated that any impact on behaviour was mediated by the consumer’s attention to and cognitive processing of the cancer warning label message.^26^ Another study examined the impact of repeated exposure to labels and showed HWLs increased knowledge and decreased intentions to consume alcohol, but despite the novelty of the repeated exposure approach, its limitation with regard to ecological validity was its implementation with online stimuli rather than labels on containers in a real world setting.^20^ This study also showed health warning impact on negative emotions, and that lower exposure to HWL led to more positive emotions associated with the product, indicating the possible role of HWL in decreasing the marketing appeal of alcohol packaging.^20^ In the European context, a recent online experiment evaluating the impact of cancer warnings showed that a brief one-time exposure to a cancer warning significantly increased consumer knowledge of alcohol causing cancer.^27^ However, the brief exposure to the stimuli, the online setting and the short follow-up period limited the generalisability of the findings on the possible longer-term impact of labels in a real-world context.

These studies also echo a recent perspective in the WHO report that the role of alcohol labels is not limited to behaviour change but can have alternative aims such as increasing knowledge on alcohol harms, increasing support for other alcohol policies or decreasing product appeal.^3^ However, at this point, there is a lack of a more systematic examination of the impact of labels on alternative outcomes, as well as an examination of the impacts of real-world exposure to labels. To date, one real world study showed the relationship between knowledge increase and policy support,^24^ while the product appeal has not been investigated in such labelling studies.

Another aspect that has not been studied in the alcohol field is the importance of the label positioning—in the field of nutritional labelling, the effectiveness of front-of-pack labelling has been well established,^28^ but there has been no empirical research into the role of on alcohol labels despite theoretical considerations that favour the front positioning due to greater visibility.^29^ Finally, while an aspect of methodology rather than an aspect of effectiveness assessment, the possible testing effects while examining knowledge will be studied, as other pilot studies indicate that testing knowledge prior to the intervention might impact its salience and thus any effects could not be attributable solely to the intervention.^30^

### Study objective

To address the gaps in the literature, the present study aims to experimentally test the impacts of a cancer warning label on alcohol containers relative to a control label on cognitive and behavioural outcomes, as well as possible alternative roles of labels, in a real-world setting. More specifically, the study will compare the real-world effects of a label containing a message on alcohol causing cancer, which has been shown to previously already be effective in an online experiment,^27^ with a control label promoting responsible drinking, a message often used voluntarily by the alcohol producers.^31^ This active comparator has been chosen in order to reflect the messaging used in the existing practice. Additionally, the study will investigate whether the positioning of the label on the front principal panel or back panel of the container has any additional influence.^28^

The primary objective of the study is to examine the real-world effect of the label content (responsibility vs cancer message) and its positioning (front vs back) on knowledge of alcohol causing cancer. The secondary objectives of the study are to examine the real-world effect of the label content and its positioning on risk perception, emotional response to the label, behavioural intentions and self-reported alcohol consumption behaviour, as well as product appeal and support for alcohol policies. The selected outcomes are in line with the theoretical models of behaviour change,^32 33^ but consider also the alternative roles of alcohol labels.^3^ Additionally, the study aims to explore whether the labels have the same effect on different population sub-groups, including by gender, age and alcohol consumption levels, and whether there is an effect of pre-measurement on the primary outcome to adjust for learning effects in the study.

### Hypotheses

Based on the available literature, we postulate the following hypothesis for the primary outcome:

- H1: At the 1-month follow-up, knowledge of alcohol causing cancer (operationalised as a proportion of participants correctly identifying breast and colon cancers, the types of cancer mentioned in the message) will be higher among the participants who receive the label displaying a cancer message compared with those who receive the label displaying a responsibility message, regardless of the label positioning.

Additionally, we postulate the following hypotheses for the secondary outcomes:

- H2: At the 1-month follow-up, perceptions about health and cancer risks associated with alcohol consumption will be higher among the participants who receive a label containing a cancer message compared with those who receive a label containing a responsibility message, regardless of the label positioning.
- H3: At the 1-month follow-up, intentions to cut down on drinking will be higher among the participants who receive a label containing a cancer message compared with those who receive a label containing a responsibility message, regardless of the label positioning.
- H4: At the 1-month follow-up, self-reported alcohol consumption will be lower among the participants who receive a label containing a cancer message compared with those who receive a label containing a responsibility message, regardless of the label positioning.
- H5: At the 1-week follow-up, negative emotional responses to the label will be stronger among the participants who receive a label containing a cancer message compared with those who receive a label containing a responsibility message, regardless of the label positioning.
- H6: At the 1-week follow-up, the product appeal of the beverages will be lower among the participants who receive a label containing a cancer message compared with those who receive a label containing a responsibility message, regardless of the label positioning.
- H7: At the 1-month follow-up, overall support for alcohol policies considered as ‘best buys’ (ie, increase in excise taxation to increase prices, availability restrictions and marketing bans) will be higher among the participants who receive a label containing a cancer message compared with those who receive a label containing a responsibility message, regardless of the label positioning.

In addition to the confirmatory hypotheses, we will explore the following potential effects, for which there is less clear evidence in the literature:

- E1: Are there any differences in the effects of label on knowledge, risk perception, intention and self-reported alcohol consumption between the first and second follow-up?
- E2: Does the label positioning interact with the effects of the label content in the impact on primary and secondary outcomes?
- E3: Do the effects of label content and positioning vary across population sub-groups?
- E4: Is there a testing effect as participants are exposed to a question measuring knowledge of alcohol causing cancer at baseline and two follow-ups?

## Methods and analysis

### Study design

**Figure 1:**
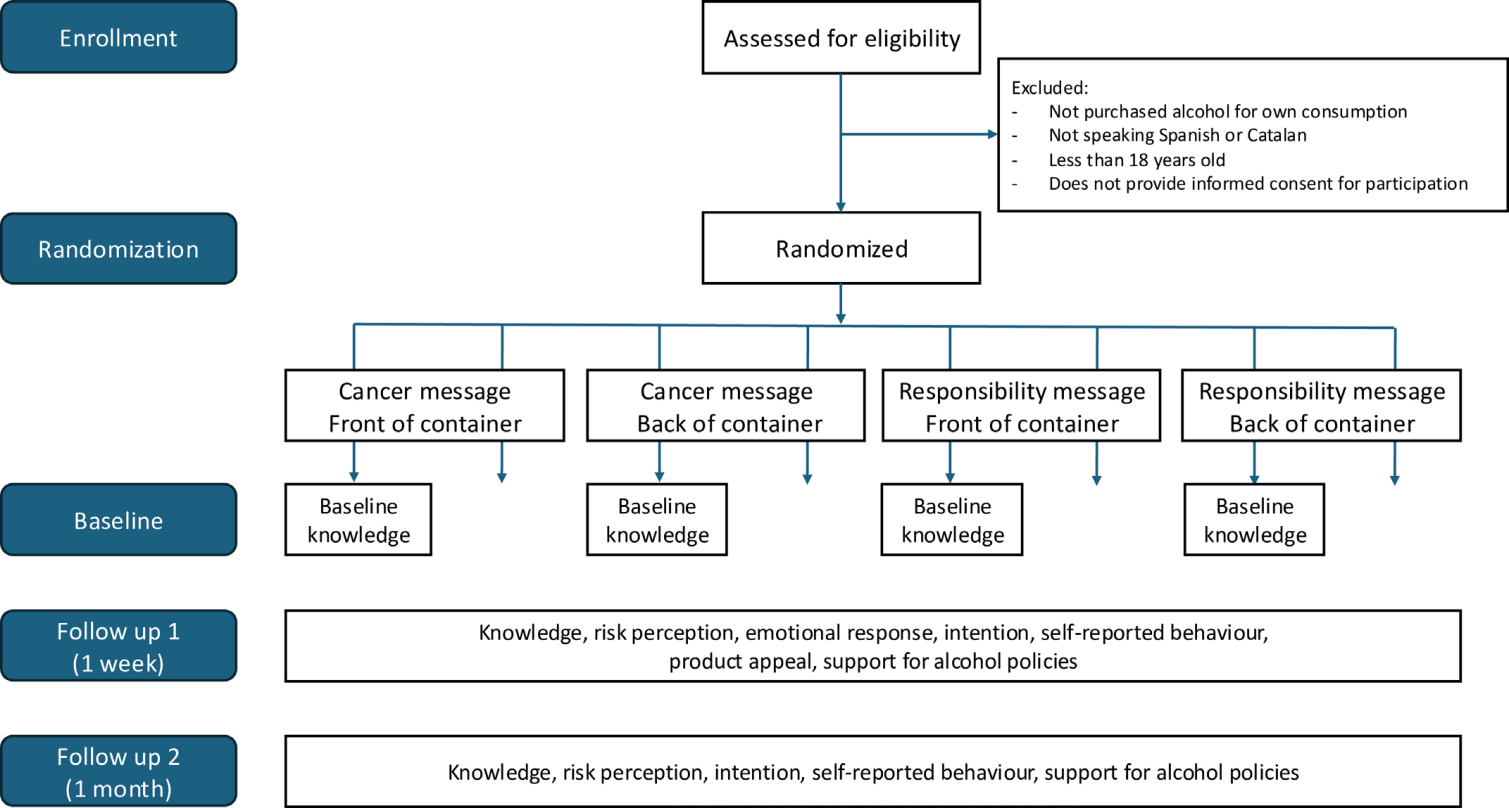
Study flowchart.

This study will employ a 2×2×2 eight-group Solomon study, with parallel design and equal allocation ratio. The three main effects being analysed are (i) label content (cancer message, responsibility message), (ii) label positioning (front of container, back of container) and (iii) pre-test for the primary outcome (present or absent). The study flowchart is presented in figure 1, with the SPIRIT participant timeline available in the online supplemental material 1.

The selected factorial design will allow us to examine the main effects of label content and label positioning, as well as their interaction. The inclusion of the pretest for the primary outcome only for half of the sample will allow investigation of possible testing effects hypothesised in similar studies,^30^ and the primary and secondary outcomes will be measured at two time points after the end of the intervention.

### Setting

This study will take place in locations outside supermarkets across different neighbourhoods in Barcelona, Spain. According to 2021 data, 78.7% of men and 55.2% of women across Barcelona reported consuming alcohol in the past 12 months.^34^ Within Barcelona, the selection of the neighbourhoods in which recruitment will be conducted will be based on the Gross Disposable Household Income index of the neighbourhood from the Health and Policy Impact Observatory of the Barcelona City Council.^35^ This is a synthetic index based on tax data at the census section level and presented in the form of a ratio between the income of the area and the average value for Barcelona as a whole. The higher the value of the index, the greater the economic capacity. Neighbourhoods from the top, middle and bottom quintile according to the index will be included to represent the different socioeconomic strata in the city. Within each of the selected neighbourhoods, the supermarket locations will be sampled based on the information provided in the store register from the Barcelona City Council. The location of the recruitment will be either on the supermarket premises after the check-out or on the street outside the supermarket, depending on the location availability and the permissions of the supermarket chains.

### Participants, sample selection and recruitment

To be eligible to participate in the study, the participants will have to be of the legal drinking age in Spain (18 years or older), having just purchased at least one alcoholic beverage (alcohol by volume ≥ 1.2%) in the sampled supermarket for their own consumption. They will have to speak Catalan or Spanish and provide informed consent to have labels applied to their alcohol purchase and to be contacted for completing the follow-up questionnaires.

Participants will be approached after the supermarket check-out or on the street outside the supermarket by a trained market research interviewer. Participants will be approached systematically (each person passing the predetermined landmark after the check-out or exiting the supermarket while the researcher is available (ie, not engaged in data collection with another participant)) and asked if they would be willing to respond to a few questions determining their eligibility to participate in a study in which they would have to complete three short questionnaires. At that point, they will also be informed that participating in the study involves monetary reimbursement. In case they meet the eligibility criteria, they will be given the full information on the study and asked whether they would be willing to participate. If there are several people approaching in a group, the person on the left side will be approached first.

The recruitment will be ongoing until a sufficient sample size at follow-up, as described in the section on the Sample size below, will be collected. Quota sampling will be applied, with cross-cell quotas to ensure a minimum of 10 participants for each combination age group (18–34, 35–54, 55 or older), gender (men or women) and educational attainment level (secondary education or lower or tertiary education). The recruitment times will depend on the most productive times at the location to maximise the recruitment rate.

Before the commencement of the study, the approach will be piloted in three locations with up to 20 participants.

### Data collection

Data will be collected using surveys at three time points, baseline or pre-intervention, 1-week follow-up post-intervention and 1-month follow-up post-intervention. After recruitment, consent and providing e-mail and phone numbers for follow-up measurements, the participants complete a brief 5 min survey, before the interviewer affixes the label sticker for each of the alcohol containers they have purchased for their consumption. The full procedure is completed in less than 10 min. The participants are contacted in 7 days for a first follow-up (10 min survey), and in 1 month for a second follow-up (5 min survey). The baseline survey will be completed in person by the participants on a tablet, and the follow-up surveys will be completed online via the participant’s device (mobile phone, tablet, computer). To increase participant retention, the participants will receive a follow-up e-mail if they do not respond after the first email prompt. If there is no response after two emails, the research team will call participants to conduct the survey via telephone. For the two follow-up surveys, participants will have 7 days to complete the online survey.

Participants will receive compensation in the form of vouchers after each completed follow-up questionnaire (10 Euro after the first follow-up questionnaire, and 25 Euro after the second follow-up questionnaire). Participants will receive via e-mail, SMS or Whatsapp a digital voucher that can be used at major retail establishments.

All data collection will be administered between April 2025 and July 2025.

### Randomisation and blinding

Randomisation to the condition will be done by the survey programme as part of the baseline survey administered by the market research company. The randomisation will be performed using a built-in randomiser function executed through the online platform through generating random numbers immediately after the participant signs the informed consent. The interviewer, however, will only have the allocated group displayed at the end of the baseline questionnaire, just before having to apply the sticker to the alcohol container. Due to its nature, the intervention will not be concealed from the participants and the interviewers. The participants, however, will be blinded to the label condition, not randomly assigned to them, and the labels will have the same design in all four conditions.

### Intervention

**Figure 2:**
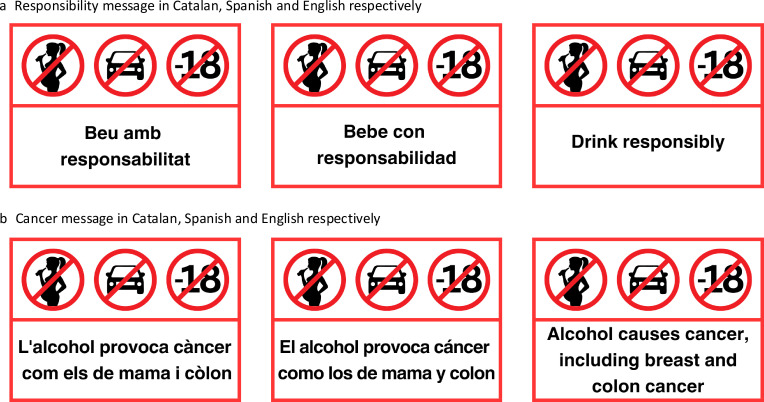
The intervention material (cancer and responsibility message) in Catalan, Spanish and English language.

Cancer message will be modelled in content based on the Yukon study as it has been pretested in Canadian (real world) and European context (online experiment).^27^ The ‘Drink responsibly’ message will reflect the wording commonly used by alcohol producers in the local context. Both messages will be available in Spanish and Catalan and participants will be able to choose the language of the questionnaires and intervention. Apart from the text, the label will be designed consistent with science-informed elements for effective product warning labels (contrasting background with clearly demarcated borders, use of colour red and use of pictograms) that will be equal for both messages in order to ensure that any result differences can be attributed to the studied variables (content and positioning of the message). Examples of labels to be tested can be found in figure 2. The size of the sticker will be approximately 5×7 cm.

The sticker with one of the two messages will be applied by the trained market research interviewer to either the front or back of the container, depending on the outcome of the randomisation, after the participant completes the baseline survey. It will be applied to all the alcoholic beverages with an ABV of 1.2% or above (legal definition of alcohol in EU and Spain as it’s Member State^36^) that the participant purchased on that supermarket visit for their consumption. This represents all beer, wine, spirit, cider, ready-to-drink mixes and other alcohol type containers. Small single serving containers (50–100 mL) will not be labelled, in case the size of the sticker is too large for the size of the container. In all cases, the label will be positioned horizontally and applied to the bottom third of the beverage. The participants will be asked not to remove the sticker once it’s placed on the alcohol container.

The length of the exposure to the health message on the label will depend on how and when the participant consumes the purchased beverages. Thus, the baseline questionnaire will contain questions on the expected amount of exposure (number of labelled containers, and participant’s plans on consumption of the containers) and in the follow-up questions, we will aim to ascertain the extent of the exposure by asking participants to provide information on the number of initially labelled containers that have either partially or fully been consumed.

### Outcome measures

#### Primary outcome

Knowledge of alcohol causing cancer will be measured in two steps. The first question, ‘Which of the following diseases and conditions does alcohol consumption increase the risk of? (select all that apply)’, with the possible responses including: ‘Cancer’, ‘Heart disease’, ‘Respiratory disease’, ‘Alcohol use disorder’, ‘Injuries’, ‘Diabetes’ and ‘Liver disease’ listed in random order, and ‘I don’t know’ and ‘None’ as exclusive options. If participants select ‘Cancer’ as an outcome, a follow-up question will ask: ‘Which of the following cancers do you think drinking alcohol increases the risk of? (select all that apply)’, with ‘Female breast cancer’, ‘Liver cancer’, ‘Colon cancer’, ‘Skin cancer’ and ‘Oral cancer’, listed in random order, and ‘I don’t know’ and ‘None’ as exclusive options. The question format and options were adapted from a survey study^37^ and previously used in an online experiment on alcohol labelling.^27^ The outcome will be dichotomous based on whether the participants correctly identify the link between alcohol and two cancers mentioned in the label: breast and colon cancer, with ‘1’ in case both answers will be selected, and ‘0’ if none or one answer will be selected correctly. The questions will be posed to half of the sample at baseline and will be present in both follow-up questionnaires.

#### Secondary outcomes

##### Behaviour

Change in alcohol consumption will be measured by asking about the number of standard drinks consumed in the last 7 days, as well as whether they have foregone an alcoholic drink because they wanted to drink less, with questions adapted from previous studies.^24 26 30^ These variables will be measured at all three time points.

##### Risk perception

Perceived personal cancer risk (adapted from previous studies^17^) will be measured with the question ‘If I consume alcohol on a regular basis, I am at greater risk of getting cancer’. Perceived general health risk will be measured with the question ‘If I consume more alcohol, I am at greater risk for health harm’. Both questions will be measured on a 5-point Likert scale (strongly disagree to strongly agree), at both follow-ups.

##### Intention

Adapted from previous studies,^17^ it will be measured with the question ‘I intend to cut down on the number of alcohol units that I drink in the forthcoming month’ on a 5-point Likert scale (strongly disagree to strongly agree), at both follow-ups.

##### Emotional response to the labels

It will only be measured among those reporting in a separate question to have noticed the labels, with the question adapted from Brennan *et al*.^20^ ‘Thinking about the labels on the alcohol containers I’ve seen as part of this study, I felt: disgusted/afraid/uncomfortable/worried/excited/pleased’ with Likert answers scale from 1 to 5 (1 = ‘not at all’, 3 = ‘moderately’ and 5 = ‘very’). Emotional response will only be measured at the first follow-up.

##### Product appeal

It will only be measured among those reporting in a separate question to have noticed the labels, with three items developed for this study (‘I found the beverages less appealing after noticing the health information on the labels’, ‘The health information on the labels reduced my enjoyment of these beverages’ and ‘I felt the health information on the label detracted from the overall alcohol product appearance’) on 5-point Likert scale (strongly disagree to strongly agree) at the first follow-up.

##### Support for alcohol policies

It will be measured by listing a range of alcohol policies (eg, increasing the price of alcohol, reducing the number of outlets that sell alcohol) and asking the participants ‘To reduce the problems associated with drinking alcohol, to what extent would you support or oppose the following alcohol-related policies?’ on a scale from 1 (strongly oppose) to 5 (strongly support). The questions have been developed for this study based on previous similar investigations^38^ and will be posed at both follow-ups.

### Covariates and process variables

The covariates will be collected at baseline, including sociodemographic information such as age group, gender, educational attainment level and income level, as well as participants’ habits around reading product label use and their general alcohol consumption level, which will be measured using the AUDIT-C scale.^39^ Furthermore, the exposure questions will capture the number and types of alcohol purchases made at the supermarket on the day of the recruitment, as well as participants’ plans for consuming the purchased alcohol containers, and will be measured at baseline. The questionnaire will also contain questions on recipients’ interaction with the message: both follow-ups will contain questions on whether the participants have noticed and read the label (questions adapted from previous studies^24 26^), and the first follow-up will contain questions about engagement with the message on the label and whether the message was perceived as acceptable and relevant (adapted based on Correia *et al*^27^). Participants will also have space to leave open comments about their experiences and perceptions, which will allow them to capture any unintended and unexpected effects.

The questionnaire is added in online supplemental material 2.

### Sample size calculation

For the primary outcome of knowledge, the sample size calculation was based on a two-sided comparison of proportions, using a significance level of 0.05 and a power of 80%, to test the main hypothesis that at the 1-month follow-up, knowledge of alcohol causing cancer will be higher among participants who receive a cancer message label compared with those who receive a responsibility message label. Previous studies conducted in the EU indicated that approximately 15% of participants correctly identified female breast cancer and 39% identified colon cancer as linked to alcohol consumption.^6^ As the present outcome is defined as the proportion of participants correctly identifying both cancers mentioned in the message, a conservative baseline prevalence of 13% was used. The study was powered to detect a medium-to-large effect, corresponding to an OR of 2.5, consistent with the previous online experiment^27^ but adjusted for the longer exposure and follow-up expected to attenuate immediate effects. An OR of 2.5 with a baseline of 13% corresponds to an increase to approximately 27%. Sample size calculations were conducted in R using the *power.prop.test*() function, indicating that around 125 participants per group (ie, message type) were required, for a total of 250 participants.

For self-reported behaviour as the key secondary outcome, the factorial structure is modelled via multiple regression. Sample size was computed for an F-test of this five-predictor block under an unconditional model, considering f²=0.02 with α=0.05 and 80% power. Under these assumptions, the required total sample size is around 650 participants. Sample size calculations were conducted in R using the pwr.f2.test() function from the pwr package.

The f² = 0.02 for behavioural outcomes has been estimated to correspond to approximately a 5–10% difference in reported alcohol consumption between intervention groups. For the dichotomous behavioural measure (foregoing a drink), this translates to detecting approximately an 8 percentage point difference between groups.^30^ For standard drink consumption, this would allow the detection of a difference of approximately 1.5–2 standard drinks per week between groups.^40^

Expecting the response rate of 50% at the second follow-up (based on the conservative estimate by the market research agency), this means the initial sample size required across the four conditions is 1300 participants.

### Data management and analysis

The data collected during the study will be securely stored in the secured online systems of the market research company. To ensure integrity and confidentiality, all transfers to the data analysis team will be conducted via secured channels. We will give descriptive results for all outcomes and key influencing factors. An attrition analysis will be conducted to determine whether loss to follow-up differs significantly across experimental conditions at both the 1-week and 1-month follow-ups and whether there are systematic sociodemographic differences in participants dropping out of the study.

For the outcomes with available baseline data (knowledge of alcohol causing cancer, foregoing alcoholic drinks, number of standard drinks consumed), we will conduct an intention-to-treat (ITT) analysis to account for dropouts, assuming no outcome change from baseline among the non-responders. Sensitivity analyses will compare ITT analysis results with results of complete case analysis, as well as with ITT analysis results with outcome data imputed based on the available covariates. For all the other outcomes, only a complete case analysis will be conducted.

To evaluate the impact of HWLs on knowledge of alcohol causing cancer, logistic regression models will be used to estimate the probability of a correct response as a function of key independent variables, including label content (cancer message vs responsibility message), label positioning (front vs back of the container) and pre-test condition (pre-tested vs not pre-tested). The analysis will assess both the main effects of the intervention and the pre-test, as well as potential interaction effects between these variables, allowing for an evaluation of whether the pre-test condition modifies the impact of the labels. To ensure the robustness of findings, multiple sensitivity analyses will be conducted. First, additional models will be fitted separately for post-test-only groups and pre-test/post-test groups to isolate the independent effects of the intervention and the pre-test condition. Second, sensitivity analyses will be done with (a) only the answer to the first knowledge question as an outcome (comparing participants who have selected cancer as a consequence of alcohol use with those who have not, regardless of their answer to the follow-up question on specific cancers) and (b) consideration of the incorrect answer on the specific cancer question (selection of ‘Skin cancer’ as an answer). Third, sociodemographic characteristics such as age, gender and education will be tested for their potential influence on the outcome. If significant interactions between demographic characteristics and the intervention are detected, further exploratory analyses will be conducted to understand how these interactions influence the effectiveness of the intervention.

Secondary outcomes measured on a five-point Likert scale will be analysed using linear regression models, treating the variables as continuous to facilitate interpretation of mean differences between intervention groups. The secondary outcome measuring the number of standard drinks will be tested for continuous versus count distribution and analysed using linear regression or Poisson regression, respectively. In the case of a count distribution exhibiting overdispersion, a negative binomial regression model will be applied. For binary outcomes, as well as for ordinal variables dichotomised a priori, logistic regression models will be used. As before, the model will include label content (cancer message vs responsibility message), and label positioning (front vs back of the container) as independent variables. To account for potential confounding effects, sociodemographic variables, including age, gender and education, as well as variables related to alcohol purchase and consumption (AUDIT-C score, number of labels applied) will be tested for their influence on each secondary outcome. If significant, these variables and their interactions with the intervention will be included in the final model.

The results will be reported as ORs for logistic regressions or regression coefficients (β) for linear regressions, together with their 95% CIs, to quantify the strength and direction of the associations. All analyses will be conducted separately for outcomes measured at the 1-week follow-up and 1-month follow-up, for outcomes assessed at both time points. This will allow us to assess the immediate and cumulative effects of exposure to HWLs over time, providing a comprehensive evaluation of their effectiveness in enhancing knowledge about alcohol-related health risks. All analyses will be conducted using R, assuming a significance level of α=0.05.

Open-ended responses about participants’ reactions to warning messages will be analysed using a thematic analysis approach.

### Monitoring

No independent data monitoring committee will be established given the low-risk nature of this brief behavioural intervention study. The market research company responsible for data collection will conduct allocation checks and data quality verification, as well as providing weekly reports on recruitment rates and data completeness to the research team for review.

### Public involvement

The protocol was developed in collaboration with the Public Health Agency of the Local Government of Catalonia, Spain (Generalitat Catalunya). The protocol was presented and discussed with members of the WHO EVID-ACTION Alcohol Network to include their perspectives on the study design and methodology. No other public representatives have been involved.

### Ethics and dissemination

#### Ethical considerations

The study has been approved by the Ethical Committee for Research with Medicines (CEIm) IDIAP Jordi Gol (24/228-P) and the Ethical Research Committee of the WHO (ERC.0004213).

Participants will provide informed consent ahead of participation in the study (consent available in online supplemental material 3). Before participation, all participants will be given the participant information sheet and have time to read it and ask any questions before signing the informed consent. They will be informed they are able to decline the participation in the study or withdraw from the study at any time without providing a reason.

To ensure privacy and confidentiality, participants will also be informed about the collection and processing of their data and assured their data will not be securely treated and not identifiable in any published results. Additionally, participants will be informed about their rights to request a copy of a collection of all the personal data and amend or delete any data they deem necessary.

Although the study does not involve direct risks to participants, some individuals may experience emotional distress when exposed to the warning that communicates ways in which alcohol harms their health or become distressed about their drinking behaviour. To mitigate this, they will be provided with sources of additional information and support in study debrief after completing the last follow-up questionnaire.

### Data sharing

The dataset will be available following the open data-sharing practices and will be available on the Figshare repository (https://figshare.com/) once the article with the main results is published. The interested researchers will be able to access and download the data directly through the platform, without requiring any permissions. The article, including the dataset, will be shared under a non-commercial licence (eg, Creative Commons Attribution-NonCommercial (CC BY-NC)), allowing others to use the data for research, educational or other non-commercial purposes, but not for commercial advantage or monetary gain.

### Dissemination of the study results

The findings of this study will be disseminated through multiple channels to ensure broad reach and impact. The results will be published in peer-reviewed journals and shared through WHO/Europe and partner organisations’ social media platforms. Additionally, they will be presented to relevant stakeholders via the EVID-ACTION project network, which includes EU Member States’ alcohol focal points from health ministries, health professionals, civil society organisations, young people, early career researchers and people with lived experience. The study results will also form part of the report on health warnings developed by the WHO Regional Office for Europe as a deliverable for the European Commission (the study funder) and presented as requested by the funder in the EU fora. At the national and regional levels in Spain, the results will be shared through the already established collaborations with the alcohol focal points from the Ministry of Health in Spain, and the partners from the Local Government of Catalunya, ensuring that findings contribute to ongoing policy discussions and public health initiatives. Furthermore, when signing informed consent, participants will be given the option to opt in to receive study findings once available.

## Discussion

Health labelling of alcoholic beverages is increasingly discussed as an important policy option to raise awareness of alcohol-related risks and reduce alcohol-related harm. The Global alcohol action plan 2022–2030 to strengthen the implementation of the WHO Global Strategy to Reduce the Harmful Use of Alcohol^41^ encourages the Member States to consider appropriate consumer protection measures through the development and implementation of labelling requirements for alcoholic beverages. In the European context, the momentum is building: in Ireland, mandatory health warnings including cancer warnings have been included in the 2018 Public Health Alcohol Act^42^ and the Norway Directorate of Health was instructed by the Norwegian Minister of Health and Care to assess how alcoholic beverages can be equipped with health warnings.^43^ A proposal for health warnings on alcoholic beverage products has also been included in the implementation roadmap of Europe’s Beating Cancer Plan.^44^

On the other hand, there is limited evidence on the impact of health warnings in naturalistic settings; most studies to date have been conducted in online or lab environments. This study will be the first one in Europe to assess how people engage with health information provided on actual alcoholic beverages they are consuming. The study will also provide invaluable evidence on whether the knowledge increase that has previously been demonstrated in the short term also persists in the longer term, and whether engagement with the label can also lead to a decrease in alcohol consumption. Furthermore, the study will provide an indication of the potential merits of front-of-package labelling, and the potential impacts of health warnings on alternative outcomes such as product appeal and support for alcohol policies. This information will be helpful for policymakers who are considering mandating health warnings on labels in their jurisdiction.

### Study limitations

#### Exposure heterogeneity and duration

Although the exposure will likely be longer than the one-time brief exposure used in most online and lab-based labelling studies to date, we can still expect heterogeneity of exposure depending on the amounts purchased and the length of consumption. For this reason, we are collecting data on the label exposure in the baseline and follow-up questionnaires. Another limitation is that participants will only receive the intervention at one time, with a follow-up period of a maximum of 1 month. While we mention that the study will provide evidence on whether knowledge increases are sustained over the longer term, this 1-month follow-up period is relatively short and may not capture truly long-term effects or habituation effects (although compared with most existing alcohol health warning studies, 1-month follow-up can still be considered relatively long term). Longer-term research would be needed to assess whether warning effectiveness diminishes over time as people become accustomed to seeing them repeatedly. Additionally, alcohol products consumed by the participant during the study period other than those purchased at the supermarket on the day of recruitment will not be labelled, which makes any estimated effects more conservative.

#### Point-of-sale recruitment and potential biases

We acknowledge that recruiting participants outside the stores could introduce selection bias, as those who agree to participate may differ systematically from the general population in ways that affect their response. As the study has experimental design, however, our aim is not to collect data from a representative sample, and this recruitment method provides ecological validity and captures real purchase behaviour, representing a trade-off between contextual biases and real-world applicability. Additionally, participants are informed of the reimbursement before providing consent, which may influence participation decisions, with financial incentives attracting participants who differ systematically in socioeconomic status, motivation or drinking patterns from those who would participate without payment. To assess this possibility, we will be collecting basic sociodemographic data also by the eligible potential participants who do not want to participate in the study. Recruiting individuals at the point of sale also introduces several other potential biases. First, point-of-sale timing may induce social desirability bias and defensive processing at baseline, as participants may feel less comfortable disclosing heavy or risky drinking patterns, especially if they are with others. Assessing knowledge of health risks immediately after purchasing alcohol may lead participants to suppress or downplay those risks compared with assessments conducted at other times or in other contexts, potentially resulting in higher defensive processing at baseline. This timing difference creates a potential mode effect: any increases in risk knowledge or awareness from baseline to follow-up could be partially attributable to reduced defensiveness in the follow-up context rather than solely to the label intervention itself. This could artificially inflate the observed differences between baseline and follow-up scores. Second, point-of-sale recruitment may lead to time pressures, increased cognitive load or distractions, which may impact baseline survey responses. Third, because labels are affixed to containers post-purchase, the opportunity for labels to influence on-the-spot purchasing decisions is excluded. While our split-sample design partially addresses these concerns, it cannot fully eliminate them. Results should be interpreted considering potential cognitive dissonance effects immediately post-purchase and the possibility that observed changes may reflect both the intervention effect and mode effects related to the timing and context of assessment. Future research might benefit from alternative baseline timing (e.g. recruitment before entering the store or delayed baseline assessment) to disentangle these effects.

#### Interviewer effects and demand characteristics

Participants will observe the interviewers affixing labels to their containers, which will likely prompt them to notice and read labels to a greater extent than if they had purchased beverages with already affixed labels. This heightened attention, combined with awareness of being in a health-related study, may create interviewer demand effects whereby participants respond in specific ways during follow-ups based on their understanding of the study’s purpose. While this will be the case in all conditions and thus is less likely to influence observed differences between label types, the overall effect sizes may be different compared with what would occur with mandatory labelling or organisational implementation where labels are already affixed at purchase. Any discovered study effects are thus likely to be conservative in terms of real-world implementation but may overestimate awareness and attention compared with naturalistic exposure.

#### Label design comparisons

The study does not contain a group that would be completely without exposure to any label. The decision to include a ‘practice as usual’ group was made to replicate the labels based on alcohol producers’ commitments, although with a strengthened design that makes labels more visible. Thus, we will not be able to fully ascertain the impact of differences of design on the label and its contribution to the outcomes, as all labels will feature the same design elements.

#### Self-report bias in exposure data

While we are collecting data on label exposure duration and attention, social desirability bias may influence these self-reported responses, potentially leading to overestimation of label engagement.

## Supplementary material

10.1136/bmjopen-2025-103464 online supplemental file 1

10.1136/bmjopen-2025-103464 online supplemental file 2

10.1136/bmjopen-2025-103464 online supplemental file 3
