# Supplementary material for: Impacts of alcohol health warning labels in a real-world setting: protocol for a randomised controlled trial among supermarket customers in Barcelona
Source: BMJ Open. 2026 Jan 21;16(1):e103464. doi: 10.1136/bmjopen-2025-103464 (PMC12829369; doi:10.1136/bmjopen-2025-103464)
Supplement: online supplemental file 1 [file bmjopen-16-1-s001.docx]

SPIRIT 2025 Participant Timeline

| **STUDY PERIOD** | **Enrollment Day 0** | **Allocation**  **Day 0** | **Post-Intervention** | **Close-out** |
| --- | --- | --- | --- | --- |
| TIMEPOINT | Screening | Baseline | Week 1  (+7 days) | Month 1  (+7 days) |
| ENROLLMENT: |  |  |  |  |
| Eligibility screening | **X** |  |  |  |
| Informed consent | **X** |  |  |  |
| Randomization |  | **X** |  |  |
| INTERVENTIONS: |  |  |  |  |
| Cancer message (Front) |  | → | → | → |
| Cancer message (Back) |  | → | → | → |
| Responsibility message (Front) |  | → | → | → |
| Responsibility message (Back) |  | → | → | → |
| ASSESSMENTS: |  |  |  |  |
| Knowledge |  | **X**  **(half sample)** | **X** | **X** |
| Risk perception (general health harm, cancer) |  |  | **X** | **X** |
| Emotional response |  |  | **X** |  |
| Intention |  |  | **X** | **X** |
| Self-reported behaviour |  | **X** | **X** | **X** |
| Product appeal |  |  | **X** |  |
| Support for alcohol policies |  |  | **X** | **X** |

**Legend:**

X = Assessment conducted at this timepoint

→ = Continuous exposure to intervention message (depending on number of labelled products and speed of consumption)

Empty cell = No activity at this timepoint
