# Supplementary material for: Impacts of alcohol health warning labels in a real-world setting: protocol for a randomised controlled trial among supermarket customers in Barcelona
Source: BMJ Open. 2026 Jan 21;16(1):e103464. doi: 10.1136/bmjopen-2025-103464 (PMC12829369; doi:10.1136/bmjopen-2025-103464)
Supplement: online supplemental file 2 [file bmjopen-16-1-s002.docx]

# **Screener**

read to ALL

Good morning/ afternoon, I am _______, interviewer at Ipsos. We are conducting a study in which you could earn up to 35 eur to complete three short questionnaires, and I would like to ask you a few brief questions to see if you have the characteristics we are looking for.

ask ALL

**S1.** Have you bought any alcoholic beverages today?

*[note for the interviewer training: in case the person does not understand because they don’t speak the language, confirm that by asking the second question, and assign answer “No”]*

*[note for the interviewer training: in case the participant asks what is considered as alcoholic beverage, the definition is 1.2% or more alcohol by volume]*

[single answer]

| 1 | Yes |  |
| --- | --- | --- |
| 2 | No | terminate |
| 3 | Refused to give answer | TERMINATE |

ask ALL

**S1.b.** Are you going to consume any of these beverages, either alone or sharing them?

*[note for the interviewer training: in case the person purchased the alcohol for household but not for themselves, this still counts as a “No”]*

*[note for the interviewer training: we consider as a “Yes” persons who are going to drink the alcoholic beverage on that moment and persons who use alcohol to cook]*

*[note for the interviewer training: In case there will be more of cases where it is not clear whether to include them or not, it would be better to include them in the survey and make a note at the end in* **COMMENT_INTERVIEWER]**

[single answer]

| 1 | Yes |  |
| --- | --- | --- |
| 2 | No | terminate |
| 3 | Refused to give answer | TERMINATE |

ask ALL

**S2.** Do you speak Spanish or Catalan?

[single answer]

| 1 | Yes |  |
| --- | --- | --- |
| 2 | No | terminate |

ask ALL

**S3.** Are you 18 years or older?

[single answer]

| 1 | Yes |  |
| --- | --- | --- |
| 2 | No | terminate |

read to ALL

We are looking for participants for study conducted by the World Health Organization in collaboration with the Local Government of Catalonia.

The study aims to assess how health-related information on alcohol labels affects people's perceptions and behaviours.

Participants must be 18+ years old, have purchased an alcoholic beverage, speak Catalan or Spanish, and consent to participating and having stickers put on their purchased beverages. Your participation is entirely voluntary. You do not have to answer any question in this survey, nor do you have to complete the entire survey. If you choose to stop, your answers will be discarded and will not be used.

You will have to complete a short questionnaire about your background and drinking habits. Then, the alcoholic beverages you purchased will be affixed with health information stickers. Finally, you would have to complete short follow-up surveys sent via email after one week and one month.

The time commitment is 5 minutes for the initial questionnaire, 10 minutes for the first follow up survey and 5 minutes for the second follow up survey. If you complete the first follow up survey, you will receive a gift voucher worth 10 EUR, and if you complete second follow up survey, you will get gift voucher for 25 EUR. In total, if you complete the 3 questionnaires you will receive 35 EUR.

Your personal data will be kept confidential and secure, in line with European and national regulations. You can withdraw at any time.

ask ALL

**S4.** Are you interested in participating in the study after hearing the key information?

[single answer]

| 1 | Yes |  |
| --- | --- | --- |
| 2 | No | terminate |

[here will begin the questionnaire for the individuals that have agreed to participate in the survey, S1, S1b, S2, S3 and s4 wil be have an imputed value (1 for “yes”]

**SAMPLE**

[create an internal variable that randomly assignate a sticker tipology]

| 1 | Label cancer – Front – Q11/Q12 |
| --- | --- |
| 2 | Label cancer – Back – Q11/Q12 |
| 3 | Label responsibility – Front – Q11/Q12 |
| 4 | Label responsibility – Back– Q11/Q12 |
| 5 | Label cancer – Front – NOT Q11/Q12 |
| 6 | Label cancer – Back – NOT Q11/Q12 |
| 7 | Label responsibility – Front – NOT Q11/Q12 |
| 8 | Label responsibility – Back – NOT Q11/Q12 |

ask if s1b=1 and S2=1 And s3=1 and s4=1

**S6.** In which language would you prefer to be interviewed?

[single answer]

| 1 | Spanish |  |
| --- | --- | --- |
| 2 | Catalan |  |

*Note for the interviewer: Please check if the participant has any questions after the initial explanation of the study*

ask if s1b=1 and S2=1 And s3=1 and s4=1

*Note for interviewer: Please give the table to the respondent so they can read and sign the informed consent*

**VOLUNTARY ACCEPTANCE OF PARTICIPATION IN THE RESEARCH PROJECT**:

I freely agree to participate in the project: "Impact of providing information on the alcoholic beverage labels in a real environment”

I affirm that:

- If I needed to, I was able to ask questions about the study.
- I understand that participation is voluntary.
- I understand that participation consists of answering questions in a survey at three different times and receiving stickers with health information on the alcoholic beverages purchased.
- I understand that the personal data (e-mail and the phone number) provided below will only be used for purposes of the study follow-up and will not be shared with any third parties
- I understand that I can withdraw from the study:
  1. When I wish,

1. Without having to give explanations

In accordance with the provisions of Regulation (EU) 2016/679 of the Parliament European and the Data Protection Council of April 27 (RGPD) and the Law organic 3/2018, of December 5, on data protection and guarantee of rights digital, I declare to have been informed of my rights, on the purpose of collection of my data and recipients of the information

**CONSENT.** I voluntarily agree to participate in the project:

[single answer]

| 1 | Yes |  |
| --- | --- | --- |
| 2 | No | terminate |

Please give the table to the interviewer

[new screen]

*Note for interviewer: explain that the purpose of collecting e-mail address and phone number is to contact them for the follow-up surveys, sending the vouchers if eligible, and (if selected) sharing the study results with them. E-mail and phone number will not be used for any other purpose.*

[ask all]

First name (not surname):_______________________________________

[masking so it only can intro a correct mail]

E-mail:_______________________________________

[masking so it only can intro a correct phone number]

Phone number:________________________________

ask if s1b=1 and S2=1 And s3=1 and s4=1 and consent=1

**S5.** Are you interested in being informed about the study results?

[single answer]

| 1 | Yes |  |
| --- | --- | --- |
| 2 | No |  |

*Note for interviewer:* *Offer to the participant the participant the possibility to have more information on the study physically (as a paper to take home)*.

Ask if s4=2 OR consent=2

Could you please answer the following questions, for statistical purposes only?

*Note for interviewer: D1 to D3 self-administrated. Give the table to the respondent*

*Note for interviewer: in case the person refuses to answer the questions, fill the D1-D3 questions with 99 and D2 with visual gender.*

**D1_N.** Please indicate your age:

[single answer] [numeric answer] [range 18-120]

_____________

[recode in quota age groups]

| 1 | Under 18 years of age | D1<18. |
| --- | --- | --- |
| 2 | 18-34 years of age | D1>=18 and q1<35 |
| 3 | 35- 54 years of age | D1>=35 and q1<55 |
| 4 | 55 years of age or older | D1>=55 |
| 99 | Prefer not to answer |  |

ask ALL

**D2_N.** Please indicate your gender:

[single answer]

| 1 | Woman |
| --- | --- |
| 2 | Man |
| 3 | Non-binary / Other |
| 99 | Prefer not to answer |

ask ALL

**D3_N.** What is the highest school qualiﬁcation you have completed?

[single answer]

| 1 | Less than high school (Primaria, EGB, 1º, 2º o 3º de ESO, Bachillerato elemental) |
| --- | --- |
| 2 | High school (4º de ESO, Bachillerato, BUP, COU, CFGM, FP1) |
| 3 | Post-secondary degree or certificate (CFGS, FP2, Grado, diplomatura, licenciatura, master, doctorado) |
| 99 | Prefer not to answer |

Ask if s1=2 or S2=2 or s3=2 or s4=2 or S5=2

**COMMENT_INTERVIEWER.** Please note any comments you feel appropriate regarding the reaction of the interviewed person to the study or reasons for decline.

*Note for the interviewer training: has to be explained that we are interested in any possible observations that could help us improve the recruitment process and study implementation*

[open answer]

_______________________________________________________

# **Baseline questionnaire**

**Individuals of 18 years or older that have bought alcohol for own consumption**

show to all

Dear participant:

Many thanks for agree to participate. All your answers are anonymous and will be kept strictly conﬁdential. It will not be possible to link your individual responses to you in any way.

Your answers will help us to understand how people react to different information provided on the alcohol containers.

WHO may share the data and ﬁndings of this project, however no identifying information about participants will be presented.

ask ALL

**D1.** Please indicate your age:

[single answer] [numeric answer] [range 18-120]

_____________

[recode in quota age groups]

| 1 | Under 18 years of age | Q1<18. terminate |  |
| --- | --- | --- | --- |
| 2 | 18-34 years of age | q1>=18 and q1<35 | at least 10 |
| 3 | 35- 54 years of age | q1>=35 and q1<55 | at least 10 |
| 4 | 55 years of age or older | q1>=55 | at least 10 |

ask ALL

**D2.** Please indicate your gender:

[single answer]

| 1 | Woman | at least 10 |
| --- | --- | --- |
| 2 | Man | at least 10 |
| 3 | Non-binary / Other |  |
| 99 | Prefer not to answer |  |

ask ALL

**D2_B.** Please, indicate your biological sex:

[single answer]

| 1 | Female |  |
| --- | --- | --- |
| 2 | Male |  |
| 99 | Prefer not to answer |  |

ask ALL

**D3.** What is the highest school qualiﬁcation you have completed?

*Note for interviewer: Read possible answers*

[single answer]

| 1 | Less than high school (Primaria, EGB, 1º, 2º o 3º de ESO, Bachillerato elemental) | at least 10 |
| --- | --- | --- |
| 2 | High school (4º de ESO, Bachillerato, BUP, COU, CFGM, FP1) |  |
| 3 | Post-secondary degree or certificate (CFGS, FP2, Grado, diplomatura, licenciatura, master, doctorado) | at least 10 |

ask ALL

**D4.** How would you describe the current level of your household income?

*Note for interviewer: Read possible answers*

[single answer]

| 1 | It's enough to live comfortably |
| --- | --- |
| 2 | It's enough, but you don't have much left over |
| 3 | It's not enough, and you find it difficult to get by |
| 4 | It's not enough to live on |
| 99 | [DO NOT READ] Prefer not to answer |

show to all

The next questions are about how often you drank alcohol in the past 12 months and how much you drank usually on the days when you drank.

[new screen] read to all

We need the answers you give us about your alcohol consumption to be recorded in Standard Drinking Units. The Standard Drinking Unit (SDU) is a quick and convenient way of finding out how many grams of alcohol you have consumed, using only a table of equivalences with the amount and type of alcoholic drink consumed to calculate your consumption.

[new screen] read to all


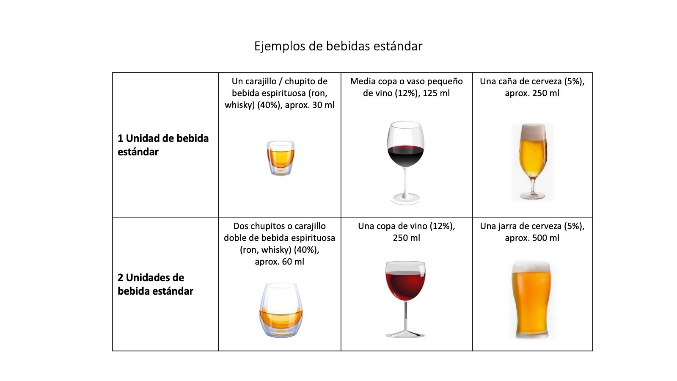
This picture, which you will find in all questions about alcohol consumption, shows the equivalences between one or two SBUs with different types and amounts of alcohol. Do you need any clarification? [Show image that explains standard drinks]

[new screen]

*Note for interviewer: Questions about alcohol consumption are self-administered (Q1-Q6). Please pass the device to the respondent so that they can answer.*

ask ALL

**Q1.** How often did you have a drink containing alcohol in the past 12 months?

[single answer]

| 1 | Never | go to Q7 |
| --- | --- | --- |
| 2 | Monthly or less |  |
| 3 | 2-4 times per month |  |
| 4 | 2-3 times per week |  |
| 5 | 4+ times per week |  |
| 99 | Prefer not to answer |  |

ask if q1>1

**Q2.** How many standard drinks of alcohol did you have on a typical day when you were drinking, in the past 12 months?

Please refer to the image below to quantify the number of drinks in Standard Drinking Unit (SDU).

[Show image that explains standard drinks]

[single answer]

| 1 | 1-2 |
| --- | --- |
| 2 | 3-4 |
| 3 | 5-6 |
| 4 | 7-9 |
| 5 | 10 + |
| 99 | Prefer not to answer |

ask if q1>1

**Q3.** How many standard drinks of alcohol did you drink over the last 7 days?

Please refer to the image below to quantify the number of drinks in Standard Drinking Unit (SDU).

[Show image that explains standard drinks.]

[single answer] [numeric answer integer] [range 0-999]

Number of standard drinks: ____

| 999 | Prefer not to answer |
| --- | --- |

ask if q1>1

**Q4.** How often have you had [SHOW TEXT ACCORDING TO D2_B: 🡪IF D2_B=2 text 6 or more / IF D2_B=1 text 4 or more] standard drinks of alcohol on a single occasion in the past 12 months?

Please refer to the image below to quantify the number of drinks in Standard Drinking Unit (SDU).

[Show image that explains standard drinks]

[single answer]

| 1 | Never |
| --- | --- |
| 2 | Less than monthly |
| 3 | Monthly |
| 4 | Weekly |
| 5 | Daily or almost daily |
| 99 | Prefer not to answer |

ask if q1>1

**Q5.** From the options below, what type of alcoholic drink do you consume most often?

[single answer]

| 1 | Beer |
| --- | --- |
| 2 | Wine (including Cava) |
| 3 | Spirits/Liquor |
| 99 | Prefer not to answer |

ask if q1>1

**Q6.** In the last 7 days, did you ever forego an alcoholic drink because you wanted to drink less?

[single answer]

| 1 | Yes |
| --- | --- |
| 2 | No |
| 99 | Prefer not to answer |

show all

Please, return the device to the interviewer.

ask ALL

**Q7.** How often do you look closely and read the information on the alcohol label when deciding about purchasing an alcoholic beverage?

*Note for interviewer: Read possible answers.*

[single answer]

| 1 | Never |
| --- | --- |
| 2 | Rarely |
| 3 | Sometimes |
| 4 | Often |
| 5 | Always |
| 99 | [DO NOT READ] Prefer not to answer |

ask ALL

**Q8.** Please note how many bottles/cans of alcohol have you purchased during your current shop at this supermarket:

[single answer by row]

[control. don’t let go to nexT question if all answers are 0]

|  | **Alcohol typology** | **Num cans / bottles** |
| --- | --- | --- |
| 1 | Beer | [numeric ANSWER] [range 0-99] |
| 2 | Wine (including Cava and sangria) | [numeric ANSWER] [range 0-99] |
| 3 | Spirits/Liquor | [numeric ANSWER] [range 0-99] |
| 4 | Pre-mixed drinks (e.g. hard seltzer) | [numeric ANSWER] [range 0-99] |
| 5 | Cider | [numeric ANSWER] [range 0-99] |
| 6 | Vermut | [numeric ANSWER] [range 0-99] |
| 7 | Other [SPECIFY] | [numeric ANSWER] [range 0-99] |
| 8 | Other [SPECIFY] | [numeric ANSWER] [range 0-99] |
| 9 | Containers too small to put sticker on | [numeric ANSWER] [range 0-99] |

ask ALL

**Q9.** How do you intend to consume these beverages?

*Note for interviewer: Read possible answers. We want to capture all the possible ways they plan to consume beverages - e.g. maybe they bought a bottle of wine they will share with a friend, but a beer that they will consume on their own.*

[multiple answers possible]

| 1 | On my own |  |
| --- | --- | --- |
| 2 | With another person |  |
| 3 | Shared with a group of people (even if they don’t share the beverage) |  |
| 4 | I will use it to cook |  |
| 5 | I don’t know yet [SINGLE ANSWER] |  |
| 6 | Will not consume it, I purchased for someone else [SINGLE ANSWER] | the survey ends and participant is informed that they do not meet the eligibility criteria to participate. |
| 7 | Other [SPECIFY] |  |
| 99 | [DO NOT READ] Prefer not to answer [SINGLE ANSWER] |  |

ask if q9=1 or q9=2 or q9=3 Or q9=4 OR Q9=5 OR Q9=7

**Q10.** When do you intend to consume these beverages?

*Note for interviewer: Read possible answers. We want to know when they plan to open and begin to consume these beverages, not if they don’t know when they will finish.*

[multiple answers possible]

| 1 | In the next hour |
| --- | --- |
| 2 | Within 24 hours after the purchase |
| 3 | Within the next week |
| 4 | Within the next month |
| 5 | Within the next year |
| 6 | I don’t know yet [SINGLE ANSWER] |
| 99 | [DO NOT READ] Prefer not to answer [SINGLE ANSWER] |

ask only to half the sample previously randomized (SAMPLE=1,2,3 or 4)

**Q11.** Which of the following diseases and conditions does drinking alcohol increase the risk of? You can select all that apply

*Note for interviewer: Read possible answers.*

[multiple answers possible] [randomize answers] [keep fixed codes 97, 98, 99]

| 1 | Cancer |
| --- | --- |
| 2 | Heart Disease |
| 3 | Liver Disease |
| 4 | Respiratory Disease |
| 5 | Alcohol use disorder |
| 6 | Injuries |
| 7 | Diabetes |
| 97 | None [SINGLE ANSWER] |
| 98 | [DO NOT READ] I don't know [SINGLE ANSWER] |
| 99 | [DO NOT READ] Prefer not to answer [SINGLE ANSWER] |

ask if Q11=1

**Q12.** Which of the following cancers do you think drinking alcohol increases the risk of? You can select all that apply

*Note for interviewer: Read possible answers.*

[multiple answers possible] [randomize answers] [keep fixed codes 6, 97, 98, 99]

| 1 | Female breast cancer |
| --- | --- |
| 2 | Liver cancer |
| 3 | Colon cancer |
| 4 | Skin cancer |
| 5 | Oral cancer |
| 6 | Other [SPECIFY] |
| 97 | None [SINGLE ANSWER] |
| 98 | [DO NOT READ] I don't know [SINGLE ANSWER] |
| 99 | [DO NOT READ] Prefer not to answer [SINGLE ANSWER] |

*Note for interviewer:* *Self-administrated. Give the table to the respondent*

ask ALL

**D5.** One last question, for statistical proposes only, could you please specify your net monthly household income:

[single answer]

| 1 | Up to €999 |
| --- | --- |
| 2 | €1,000 to €1,499 |
| 3 | €1,500 to €1,999 |
| 4 | €2,000 to €2,999 |
| 5 | €3,000 to €4,499 |
| 6 | €4,500+ |
| 98 | I don’t know |
| 99 | Prefer not to answer |

show all

Please, return the device to the interviewer.

[Based on SAMPLE VARIABLE show the sticker that has to be affixed]

*Note for interviewer. Affix the following sticker on the beverages:*

| 1 | Label cancer – Front | Sample=1 or 5 |
| --- | --- | --- |
| 2 | Label cancer – Back | Sample=2 or 6 |
| 3 | Label responsibility - Front | Sample=3 or 7 |
| 4 | Label responsibility - Back | Sample=4 or 8 |

read to ALL

I will now affix a sticker on the beverages you have purchased. Please do not remove the sticker from the items until the end of the study.

Ask all

**COMMENT.** Do you have any comments about the survey?

Note for the interviewer:

[open answer]

_______________________________________________________

read to ALL

Thank you for your participation! Within a week you will be contacted again with a short follow up survey.

Ask all

**COMMENT_INTERVIEWER.** Please note any comments you feel appropriate regarding the implementation of the study or the reaction of the participant

[open answer]

# **Follow up (1 week)**

show to ALL

This is a survey being conducted by the World Health Organization (WHO) Regional Oﬃce for Europe. It will take 10 minutes of your time.

All your answers are anonymous and will be kept strictly conﬁdential. It will not be possible to link your individual responses to you in any way.

Your participation is entirely voluntary. You do not have to answer any question in this survey, nor do you have to complete the entire survey. If you choose to stop, your answers will be discarded and will not be used.

Your answers will help us to understand how people react to different information provided on the alcohol containers.

WHO may share the data and ﬁndings of this project, however no identifying information about participants will be presented.

After completion of the questionnaire, you will be eligible to receive a gift voucher for Amazon, Cepsa, Carrefour or El Corte Inglés to the value of 10 EUR.

ask ALL

**Q1.** How many standard drinks of alcohol did you drink over the last 7 days?

We would like to have the answer in Standard drinks units. Please refer to the image below to quantify the number of drinks.

[Show image that explains standard drinks]

[single answer] [numeric answer INTEGER] [range 0-999]

Number of standard drinks: ____

| 999 | Prefer not to answer |
| --- | --- |

ask ALL

**Q2.** In the last 7 days, did you ever forego alcoholic drink because you wanted to drink less?

[single answer]

| 1 | Yes |
| --- | --- |
| 2 | No |
| 99 | Prefer not to answer |

ask if Q2=1

**Q3.** Please elaborate on the reasons for your decision

[open answer]

_______________________________________________________

ask ALL

**Q4.** Which of the following diseases and conditions does drinking alcohol increase the risk of? Please, select all that apply

[multiple answers possible] [randomize answers] [keep fixed codes 97, 98, 99]

| 1 | Cancer |
| --- | --- |
| 2 | Heart Disease |
| 3 | Liver Disease |
| 4 | Respiratory Disease |
| 5 | Alcohol use disorder |
| 6 | Injuries |
| 7 | Diabetes |
| 97 | None [SINGLE ANSWER] |
| 98 | I don't know [SINGLE ANSWER] |
| 99 | Prefer not to answer [SINGLE ANSWER] |

ask if Q4=1

**Q5.** Which of the following cancers do you think alcohol drinking increases the risk of? Please, select all that apply

[multiple answers possible] [randomize ANSWERS] [keep fixed codes 97, 98, 99]

| 1 | Female breast cancer |
| --- | --- |
| 2 | Liver cancer |
| 3 | Colon cancer |
| 4 | Skin cancer |
| 5 | Oral cancer |
| 6 | Other [SPECIFY] |
| 97 | None [SINGLE ANSWER] |
| 98 | I don't know [SINGLE ANSWER] |
| 99 | Prefer not to answer [SINGLE ANSWER] |

ask ALL

**Q6.** To what extent do you agree with the following statements?

Please indicate your answer on the scale below from 1 (strongly disagree) to 5 (strongly agree)

[single answer by row] [randomize statements]

|  |  | **Strongly disagree** | **Disagree** | **Neither agree nor disagree** | **Agree** | **Strongly agree** | **Prefer not to answer** |
| --- | --- | --- | --- | --- | --- | --- | --- |
| A | If I consume more alcohol, there is a greater risk of harm to my health. | 1 | 2 | 3 | 4 | 5 | 9 |
| B | If I consume alcohol on a regular basis, I am at greater risk of getting cancer. | 1 | 2 | 3 | 4 | 5 | 9 |
| C | I intend to reduce the number of standard drinks that I drink in the next 30 days | 1 | 2 | 3 | 4 | 5 | 9 |

ask ALL

**Q7.** Did you notice the health information that was provided on the alcoholic beverage label?

[single answer]

| 1 | Yes |
| --- | --- |
| 2 | No |
| 99 | Prefer not to answer |

read if q7=1

Please answer the following questions about the health information that was provided to you through the product label:

ask if q7=1

**Q8.** Did you read the written message provided on the alcohol container label?

[single answer]

| 1 | Yes |
| --- | --- |
| 2 | No |
| 99 | Prefer not to answer |

ask if q7=1

**Q9.** Can you repeat the written message you have read on the alcohol container label?

[open answer]

_______________________________________________________

ask if q7=1

**Q10.** Have you done any of the following as a result of encountering the information on the alcohol container label:

[MULTIPLE answer]

| 1 | Searched for more information about the risk to health or harms from drinking alcohol |
| --- | --- |
| 2 | Discussed the provided information with people around me (e.g. my partner, friends, or family). |
| 3 | Searched for more information about how I could reduce how much alcohol I drank . |
| 99 | Prefer not to answer |

ask if q7=1

**Q11.** To what extent do you agree with the following statements? Please indicate your answer on the scale below from 1 (strongly disagree) to 5 (strongly agree)

[single answer by row] [randomize statements]

|  |  | **Strongly disagree** | **Disagree** | **Neither agree nor disagree** | **Agree** | **Strongly agree** | **Prefer not to answer** |
| --- | --- | --- | --- | --- | --- | --- | --- |
| A | The health information on alcoholic containers made me think about the amount of alcohol I drink. | 1 | 2 | 3 | 4 | 5 | 9 |
| B | The health information on alcohol containers I have seen as part of this study is acceptable to be put on alcohol containers. | 1 | 2 | 3 | 4 | 5 | 9 |
| C | The health information on alcohol containers I have seen as part of this study is relevant to me. | 1 | 2 | 3 | 4 | 5 | 9 |
| D | The health information on alcohol containers I have seen as part of this study is believable. | 1 | 2 | 3 | 4 | 5 | 9 |
| E | I found the beverages less appealing after noticing the health information on the labels | 1 | 2 | 3 | 4 | 5 | 9 |
| F | The health information on the labels reduced my enjoyment of these beverages | 1 | 2 | 3 | 4 | 5 | 9 |
| G | I felt the health information on the label detracted from the overall alcohol product appearance | 1 | 2 | 3 | 4 | 5 | 9 |

ask if q7=1

**Q12.** Thinking about the labels on the alcohol containers I’ve seen as part of this study, I felt:

Please indicate your answer on the scale below from 1 (not at all) to 5 (very)

[single answer by row] [randomize items]

|  |  | **Not at all** |  | **Moderately** |  | **Very** | **Prefer not to answer** |
| --- | --- | --- | --- | --- | --- | --- | --- |
| A | Disgusted | 1 | 2 | 3 | 4 | 5 | 9 |
| B | Afraid | 1 | 2 | 3 | 4 | 5 | 9 |
| C | Uncomfortable | 1 | 2 | 3 | 4 | 5 | 9 |
| D | Worried | 1 | 2 | 3 | 4 | 5 | 9 |
| E | Excited | 1 | 2 | 3 | 4 | 5 | 9 |
| F | Pleased | 1 | 2 | 3 | 4 | 5 | 9 |

ask if q7=1

**Q13.** Would you like to provide any other comments about the health information on labels you encountered?

[open answer]

_______________________________________________________

ask all

**Q14.** To reduce the problems associated with drinking alcohol, to what extent would you support or oppose the following alcohol-related policies:

[single answer by row] [randomize statements]

|  |  | **Strongly oppose** | **Oppose** | **Neutral** | **Support** | **Strongly support** | **Prefer not to answer** |
| --- | --- | --- | --- | --- | --- | --- | --- |
| A | Increase the price of alcohol. | 1 | 2 | 3 | 4 | 5 | 9 |
| B | Reduce the number of outlets that sell alcohol. | 1 | 2 | 3 | 4 | 5 | 9 |
| C | Increase minimum age for buying alcohol. | 1 | 2 | 3 | 4 | 5 | 9 |
| D | Ban advertising of alcohol. | 1 | 2 | 3 | 4 | 5 | 9 |
| E | Limit advertising of alcohol. | 1 | 2 | 3 | 4 | 5 | 9 |
| F | Provide treatment for alcohol use disorders. | 1 | 2 | 3 | 4 | 5 | 9 |
| G | Add warning labels with information on alcohol-related harm to alcohol containers. | 1 | 2 | 3 | 4 | 5 | 9 |
| H | Implement drink driving measures. | 1 | 2 | 3 | 4 | 5 | 9 |
| I | Fund educational programmes for young people in schools. | 1 | 2 | 3 | 4 | 5 | 9 |
| J | Ban alcoholic beverages from being sold for home delivery through food delivery apps | 1 | 2 | 3 | 4 | 5 | 9 |

ask all

**Q15.** The list below contains the number of beverages you have purchased during your shopping trip while completing the baseline survey. Please adjust the numbers of how many of the bottles/cans of alcohol have you consumed (either partially or fully) since then.

If you don’t remember how many cans o bottles you have consumed, please type 999

[single answer by row]

[add a control to ensure that the new number of cans / bottles is equal or lower than the number of bottles from the baseline q’nre]

[ONLY THE TYPE OF ALCOHOL THAT WE HAVE INFORMATION FROM BASELINe QUESTIONNAIRE]

|  | **Alcohol typology** | **Num cans / bottles bought** | **Num cans / bottles** |
| --- | --- | --- | --- |
| 1 | Beer | show Q8_1 BS Q’nre | [numeric ANSWER] [range 0-99] |
| 2 | Wine (including Cava and sangria) | show Q8_2 BS Q’nre | [numeric ANSWER] [range 0-99] |
| 3 | Spirits/Liquor | show Q8_3 BS Q’nre | [numeric ANSWER] [range 0-99] |
| 4 | Pre-mixed drinks (e.g. hard seltzer) | show Q8_4 BS Q’nre | [numeric ANSWER] [range 0-99] |
| 5 | Cider | show Q8_5 BS Q’nre | [numeric ANSWER] [range 0-99] |
| 6 | Vermut | show Q8_5 BS Q’nre | [numeric ANSWER] [range 0-99] |
| 7 | [LITERAL FROM OTHER IN Q8_BS] | show Q8_6 BS Q’nre | [numeric ANSWER] [range 0-99] |
| 8 | [LITERAL FROM OTHER IN Q8_BS] | show Q8_7 BS Q’nre | [numeric ANSWER] [range 0-99] |

**Q16.** Do all the beverages still have on them the sticker provided by the researcher at the beginning of the study?

[singlE answer]

| 1 | Yes, all of them |
| --- | --- |
| 2 | No, some of them don't have it anymore |
| 3 | None of the, |
| 99 | Prefer not to answer |

Thank you for your participation! We will send you a separate e-mail with instructions on how to select and redeem your 10 EUR voucher.

In three weeks, you will be contacted to participate in 2^nd^ short follow up survey. Remember that if you finish the second follow up you will get gift voucher for 25 EUR.

# **Follow up (1 month)**

Dear participant:

This is a survey being conducted by the World Health Organization (WHO) Regional Oﬃce for Europe. It will take 5 minutes of your time.

All your answers are anonymous and will be kept strictly conﬁdential. It will not be possible to link your individual responses to you in any way.

Your participation is entirely voluntary. You do not have to answer any question in this survey, nor do you have to complete the entire survey. If you choose to stop, your answers will be discarded and will not be used.

Your answers will help us to understand how people react to different information provided on the alcohol containers.

WHO may share the data and ﬁndings of this project, however no identifying information about participants will be presented.

After completion of the questionnaire, you will be eligible to receive a gift voucher for Amazon, Cepsa, Carrefour or El Corte Inglés to the value of 25 EUR.

ask ALL

**Q1.** How many standard drinks of alcohol did you drink over the last 7 days?

We would like to have the answer in Standard drinks units. Please refer to the image below to quantify the number of drinks.

[Show image that explains standard drinks]

[single answer] [numeric answer integer] [range 0-999]

Number of standard drinks: ____

| 999 | Prefer not to answer |
| --- | --- |

ask ALL

**Q2.** In the last 7 days, did you ever forego alcoholic drink because you wanted to drink less?

[single answer]

| 1 | Yes |
| --- | --- |
| 2 | No |
| 99 | Prefer not to answer |

ask if Q2=1

**Q3.** Please elaborate on the reasons for your decision

[open answer]

_______________________________________________________

ask ALL

**Q4.** Which of the following diseases and conditions does drinking alcohol increase the risk of? Please, select all that apply

[multiple answers possible] [randomize answers] [keep fixed codes 97, 98, 99]

| 1 | Cancer |
| --- | --- |
| 2 | Heart Disease |
| 3 | Liver Disease |
| 4 | Respiratory Disease |
| 5 | Alcohol use disorder |
| 6 | Injuries |
| 7 | Diabetes |
| 97 | None [SINGLE ANSWER] |
| 98 | I don't know [SINGLE ANSWER] |
| 99 | Prefer not to answer [SINGLE ANSWER] |

ask if Q4=1

**Q5.** Which of the following cancers do you think alcohol drinking increases the risk of? Please, select all that apply

[multiple answers possible] [randomize answers] [keep fixed codes 97, 98, 99]

| 1 | Female breast cancer |
| --- | --- |
| 2 | Liver cancer |
| 3 | Colon cancer |
| 4 | Skin cancer |
| 5 | Oral cancer |
| 6 | Other [SPECIFY] |
| 97 | None [SINGLE ANSWER] |
| 98 | I don't know [SINGLE ANSWER] |
| 99 | Prefer not to answer [SINGLE ANSWER] |

ask ALL

**Q6.** To what extent do you agree with the following statements?

Please indicate your answer on the scale below from 1 (strongly disagree) to 5 (strongly agree)

[single answer by row] [randomize statements]

|  |  | **Strongly disagree** | **Disagree** | **Neither agree nor disagree** | **Agree** | **Strongly agree** | **Prefer not to answer** |
| --- | --- | --- | --- | --- | --- | --- | --- |
| A | If I consume more alcohol, there is a greater risk of harm to my health. | 1 | 2 | 3 | 4 | 5 | 9 |
| B | If I consume alcohol on a regular basis, I am at greater risk of getting cancer. | 1 | 2 | 3 | 4 | 5 | 9 |
| C | I intend to reduce the number of standard drinks that I drink in the next 30 days | 1 | 2 | 3 | 4 | 5 | 9 |

ask ALL

**Q7.** Did you notice the health information that was provided on the alcoholic beverage label?

[single answer]

| 1 | Yes |
| --- | --- |
| 2 | No |
| 99 | Prefer not to answer |

read if q7=1

Please answer the following questions about the health information that was provided to you through the product label:

ask if q7=1

**Q8.** Did you read the written message provided on the alcohol container label?

[single answer]

| 1 | Yes |
| --- | --- |
| 2 | No |
| 99 | Prefer not to answer |

ask if q7=1

**Q9.** Can you repeat the written message you have read on the alcohol container label?

[open answer]

_______________________________________________________

ask all

**Q10.** To reduce the problems associated with drinking alcohol, to what extent would you support or oppose the following alcohol-related policies:

[single answer by row] [randomize statements]

|  |  | **Strongly oppose** | **Oppose** | **Neutral** | **Support** | **Strongly support** | **Prefer not to answer** |
| --- | --- | --- | --- | --- | --- | --- | --- |
| A | Increase the price of alcohol. | 1 | 2 | 3 | 4 | 5 | 9 |
| B | Reduce the number of outlets that sell alcohol. | 1 | 2 | 3 | 4 | 5 | 9 |
| C | Increase minimum age for buying alcohol. | 1 | 2 | 3 | 4 | 5 | 9 |
| D | Ban advertising of alcohol. | 1 | 2 | 3 | 4 | 5 | 9 |
| E | Limit advertising of alcohol. | 1 | 2 | 3 | 4 | 5 | 9 |

ask ALL

**Q11.** The list below contains the number of beverages you have purchased during your shopping trip while completing the baseline survey. Please adjust the numbers of how many of the bottles/cans of alcohol have you consumed (either partially or fully) since then.

If you don’t remember how many cans o bottles you have consumed, please type 999

[single answer by row]

[add a control to ensure that the new number of cans / bottles is equal or lower than the number of bottles from the baseline q’nre]

[ONLY THE TYPE OF ALCOHOL THAT WE HAVE INFORMATION FROM BASELINe QUESTIONNAIRE]

|  | **Alcohol typology** | **Num cans / bottles bought** | **Num cans / bottles** |
| --- | --- | --- | --- |
| 1 | Beer | show Q8_1 BS Q’nre | [numeric ANSWER] [range 0-99] |
| 2 | Wine (including Cava and sangria) | show Q8_2 BS Q’nre | [numeric ANSWER] [range 0-99] |
| 3 | Spirits/Liquor | show Q8_3 BS Q’nre | [numeric ANSWER] [range 0-99] |
| 4 | Pre-mixed drinks (e.g. hard seltzer) | show Q8_4 BS Q’nre | [numeric ANSWER] [range 0-99] |
| 5 | Cider | show Q8_5 BS Q’nre | [numeric ANSWER] [range 0-99] |
| 6 | Vermut | show Q8_5 BS Q’nre | [numeric ANSWER] [range 0-99] |
| 7 | [LITERAL FROM OTHER IN Q8_BS] | show Q8_6 BS Q’nre | [numeric ANSWER] [range 0-99] |
| 8 | [LITERAL FROM OTHER IN Q8_BS] | show Q8_7 BS Q’nre | [numeric ANSWER] [range 0-99] |

**Q12.** Do all the beverages still have on them the sticker provided by the researcher at the beginning of the study?

[singlE answer]

| 1 | Yes, all of them |
| --- | --- |
| 2 | No, some of them don't have it anymore |
| 3 | None of the, |
| 99 | Prefer not to answer |

Thank you for your participation! In the next days, we will send you an e-mail about how you can select and redeem your 25-EUR voucher.

Please click **here** if you are interested in knowing more about the study and **here** if you are interested more in effects of alcohol on health.
