# Supplementary material for: Impacts of alcohol health warning labels in a real-world setting: protocol for a randomised controlled trial among supermarket customers in Barcelona
Source: BMJ Open. 2026 Jan 21;16(1):e103464. doi: 10.1136/bmjopen-2025-103464 (PMC12829369; doi:10.1136/bmjopen-2025-103464)
Supplement: online supplemental file 3 [file bmjopen-16-1-s003.docx]

**[EN translation]**

**INFORMATION SHEET FOR PARTICIPANTS**

**Project title:** Impact of providing information on alcohol labels in a real- world settings

**Project leaders:** General Directorate of Addictions, HIV, Sexually Transmitted Infections and Viral Hepatitis of Health Agency of Catalonia in collaboration with World Health Organization Regional Office for Europe

Dear participant,

We invite you to participate in this project that we are developing with the General Directorate of Addictions, HIV, Sexually Transmitted Infections and Viral Hepatitis of Health Agency of Catalonia in collaboration with World Health Organization Regional Office for Europe with the support of the European Commission. Before you decide whether you would like to participate, it is important that you understand why this study is being conducted and what is involved in conducting it. Please take some time to read the information below. Ask us if you need any clarification or if you want more information.

**Your participation in this study is completely voluntary.** You can decide whether you want to participate or not. If you decide to be part of the study, you can stop your participation at any time and get your research data removed and destroyed without giving any reason.

**Who can participate in the study?**

To be eligible to participate in the study, participants must be of legal drinking age in Spain (18 years or older), have purchased an alcoholic beverage (can or bottle of beer, bottle of wine or bottle of alcohol ) above ABV. 1.2% in the sample supermarket for own consumption, speak Catalan or Spanish and give informed consent to label their alcohol purchase with stickers and be contacted for follow-up questionnaires.

**Objectives**

The purpose of the study is to assess how exposure to health-related information on alcoholic beverage labels affects people's perceptions and behaviors. The scientific rationale is to provide evidence of the effectiveness of these warnings in promoting public health.

**Benefits**

You may learn new information about alcohol that could influence your relationship with alcoholic beverages in the future. No other direct benefits are anticipated. However, the results of the study will contribute to improving public health by helping to provide effective and acceptable health information on product labels.

**Study procedures**

As a participant, you will complete a short questionnaire asking basic questions about your background, your drinking habits and plans, as well as your knowledge of alcohol-related harm. This will take 5 minutes. The researcher will then apply stickers with health information to all the alcoholic beverages you purchased at the store visit. Afterwards, you can consume the labeled beverages in the way you want or have planned.

In a week, you will receive an email asking you to participate in a survey with follow-up questions. The survey will again ask you about your alcohol consumption, as well as alcohol-related knowledge or harm, attitudes towards alcoholic beverages and experiences with the intervention. This survey will take 10 minutes of your time.

Another survey will be sent to your email in a month, asking you again about your drinking habits and perceptions. This survey will take 5 minutes of your time.

**Possible risks and inconveniences**

No significant risk or discomfort is expected. The study implies a minimal possible risk of discomfort from being exposed to information about alcohol-related harm. Also, it takes time to participate. The study will take approximately 10 minutes of your time after the supermarket visit and an additional 15 minutes during two follow-ups.

You will also provide your personal data such as e-mail and phone number, which will be throughout the duration of the study connected with the questionnaire data through participant ID. Thus, there exists minimal risk of data breach in the systems. As described in the paragraph “Protection of personal data” below, utmost care is taken for the data to be safely stored to minimize such a risk. To mitigate this risk, several measures have been adopted.  Data will be anonymized to remove any personally identifiable information before analysis. Access to the data will be restricted to authorized personnel only, and all data transfers will be encrypted to prevent unauthorized access.

**Compensation**

You will be compensated for your study time. A separate incentive will be provided for each completed questionnaire (10 euros first questionnaire, 25 euros second questionnaire). You will receive digital vouchers via email, SMS or Whatsapp that can be used at major retail outlets.

**Protection of personal data**

In order to contact you for the study follow-up, we will require your e-mail and phone numbers. These will only be used for purposes of the study follow-up and will not be shared with any third parties.

In accordance with current European and national regulations for the protection of personal data, the personal data obtained are those necessary to cover the purposes of the study [Regulation 2016/679 of the European Parliament and of the Council of 27 of April 2016 on Data Protection and Organic Law 3/2018, of December 5, on Protection of Personal Data and Guarantee of Digital Rights]. Your name will not appear in any of the study reports, and your identity cannot be disclosed to anyone except to fulfil the objectives of the study, and in the case of a medical emergency or legal requirement. Any personal information that may be identifiable will be kept in secure conditions by the principal investigator or by an institution designated by him or her. Access to this information will be restricted to the staff of the collaborative project team of the Addictions, HIV, Sexually Transmitted Infections and Viral Hepatitis Branch and the Regional Office for Europe of the World Health Organization, appointed for this purpose or to other authorized personnel who will be obliged to maintain the confidentiality of the information. Data will be owned by WHO/Europe.

During the length of the project, data will be stored on servers at the market research company located in Spain. The systems are audited and aligned with ISO 27001 requirements, ensuring the confidentiality, integrity, and availability of the data. After the data collection completion and during the preparation of the final dataset, the unique identifier will be deleted, resulting in a dataset without any personal identifiers.

The final dataset will be fully anonymized and available following the open data sharing practices, and will be uploaded on Figshare repository (https://figshare.com/) by the principal investigator once the article with the main results is published, meaning the interested researchers will be able to access and download the data directly through the platform. Any personal or identifying data will be removed from that dataset, so your responses will not be able to be tracked to you. The article, including the dataset will be shared under a non-commercial license (CC BY-NC), allowing others to use the data for research, educational, or other non-commercial purposes, but not for commercial advantage or monetary gain. The dataset creators listed under the CC BY-NC license will include the members of the research team.

IPSOS will delete all processed data from their active systems within a maximum period of two months from delivery of the dataset to WHO/Europe at the end of the data collection period. Exception is the e-mail addresses of the participants who have expressed interest in receiving information about the study results and have consented to their email being used for this purpose. The e-mails of those participants will stay in a separate file without any other accompanying information and will be deleted after IPSOS informs them about the study results.

In accordance with the rights conferred on you by the current regulations on the protection of personal data, you can exercise the rights of access, rectification, limitation of treatment, deletion, portability and opposition, by addressing your request to the main researcher of the study or to the data protection delegate ([dpd@ticsalutsocial.cat](mailto:dpd@ticsalutsocial.cat ) ).

From the Sub-Directorate of Addictions, HIV, Sexually Transmitted Infections and Viral Hepatitis we will resolve all your doubts, complaints, clarifications, suggestions and attend to the exercise of rights via email: [drogues.salut@gencat.cat](mailto:drogues.salut@gencat.cat) , or by post at Carrer Roc Boronat, 81-95, 08005, Barcelona.

If you need more information about this study, you can contact the local principal investigator Joan Columbus Farran, Subdirectorate of Addictions, HIV, Sexually Transmitted Infections and Viral Hepatitis, [joan.colom@gencat.cat.](mailto:joan.colom@gencat.cat.%20)

**INFORMED CONSENT FORM FOR ADULT PARTICIPANTS**

**Project title:** Impact of providing information on beverage alcoholic labels in a real-world setting

VOLUNTARY ACCEPTANCE OF PARTICIPATION IN THE RESEARCH PROJECT:

I, ................................................ .......................... (name and surname of the participant) freely agree to participate in the project: "Impact of providing information on the alcoholic beverage labels in a real-world setting”

I affirm that:

- I have read the information sheet I have been given about the study
- If I needed to, I was able to ask questions about the study.
- I have received enough information about the study.
- I understand that participation is voluntary.
- I understand that participation consists of answering questions in a survey at three different times and receiving stickers with health information on the alcoholic beverages purchased.
- I understand that the personal data (e-mail and the phone number) provided below will only be used for purposes of the study follow-up and will not be shared with any third parties
- I understand that I can withdraw from the study:
  1. When I wish,

1. Without having to give explanations

In accordance with the provisions of Regulation (EU) 2016/679 of the Parliament European and the Data Protection Council of April 27 (RGPD) and the Law organic 3/2018, of December 5, on data protection and guarantee of rights digital, I declare to have been informed of my rights, of the purpose of collection of the my data and recipients of the information

Date and signature Date and signature

of the participant of the researcher

E-mail:

Phone number:

___ Please mark here if you would like to be informed about the results of the study and consent to your e-mail address being used for this purpose
