# Supplementary material for: Impacts of alcohol health warning labels in a real-world setting: protocol for a randomised controlled trial among supermarket customers in Barcelona
Source: BMJ Open. 2026 Jan 21;16(1):e103464. doi: 10.1136/bmjopen-2025-103464 (PMC12829369; doi:10.1136/bmjopen-2025-103464)
Supplement: Supplementary file 4 [file bmjopen-2025-103464.reviewer_comments.pdf]

# PEER REVIEW HISTORY

BMJ Open publishes all reviews undertaken for accepted manuscripts. Reviewers are asked to complete a checklist review form and are provided with free text boxes to elaborate on their assessment. These free text comments are reproduced below.

## ARTICLE DETAILS

### Title (Provisional)

Impacts of alcohol health warning labels in a real-world setting: protocol for a randomised controlled trial among supermarket customers in Barcelona

### Authors

Kokole, Daša; Neufeld, Maria; Correia, Daniela; Rehm, Jürgen; Ferreira-Borges, Carina; Hobin, Erin; Rovira, Pol; Segura, Lidia; Colom, Joan

---

## VERSION 1 - REVIEW

---

| | |
|---|---|
| **Reviewer** | **1** |
| **Name** | **Razvodovsky, Yury Evgeny** |
| **Affiliation** | **Grodno State Medical University** |
| **Date** | **24-Aug-2025** |
| **COI** | **None** |

---

The aims of this research project is to evaluate the longer-term impacts of a cancer warning label on alcohol containers relative to a control label on behavioral outcomes. This is well-designed study that addressed an important alcohol policy issue, which remains surprisingly underdeveloped. Methodologically there appear to be no critical weaknesses. I have no any specific comments.

---

| | |
|---|---|
| **Reviewer** | **2** |
| **Name** | **Green , Colin P.** |
| **Affiliation** | **Norwegian University of Science and Technology** |
| **Date** | **07-Sep-2025** |
| **COI** | **None** |

---

"Impacts of Alcohol Health Warning Labels in a Real-World Setting: Protocol for a Randomised Controlled Trial Among Supermarket Customers in Barcelona"

This protocol sets out a research plan to examine the effect of exposure to health warning labelling on alcohol on knowledge of health risks and health behaviors. It does so using a

randomized design where cancer warning labels or standard health information labels are applied to alcohol containers of individuals who have recently purchased alcohol.

In general, the protocol is clear, well-described, and the research design carefully considered. I have some comments that I feel should be further considered.

Comments:

1. The main set of concerns relate to statistical power:

a. The treatment is the application of the label to one alcoholic container. These can range (if I understand it correctly) from one beer, a bottle of wine, or bottle of spirits. In the case of the former two, this is likely a very short-term form of exposure. I.e. In many cases these will be consumed relatively quickly and disposed of. Would we expect then this exposure to this information to plausibly have large medium-term (2 months) effects. For instance, on page 13 treatment effects of 1.5 to 2 standard drinks a week. These seems large.

b. Related to this, I guess in some cases the alcohol is not (soley) drunk by the purchaser. As we don't know actual exposure to the message this gives the estimates an additional ITT interpretation and has implications for statistical power.

c. Response rate of 50% at second follow up. What is this based on? Is this conservative?

d. The study aims to test several hypotheses. This is discussed briefly in one sentence (multiple comparisons), but should this be part of the calculation of statistical power?

e. In general, my concern is that the study as described now may end in a set of uninformative null results.

2. Related to this are questions of external validity.

a. The underlying policy question is whether labelling changes behavior. The real-world setting would be moving to a setting where all alcohol was labelled. Here we examine the effect of labelling one drink container. I agree this is more realistic than lab-based approaches but has issues re: statistical power above.

b. A second related issue is that the act of applying the label is, as discussed, naturally known to subjects and one might be concerned about interviewer demand effects (i.e. that subjects who realize they have been subject to a health treatment respond in a specific way in follow-ups). It is stated that these types of effects are differenced out by the study design (all receive a label). It is not entirely clear that this is true. One related question – what is the status quo – is alcohol regularly labelled with something like the control label?

c. Finally, a concern in the real-world setting is that initial effects of the policy may 'wash-out' over time as people get used to the warnings. Here this type of effect (which is discussed) is different in that it represents differences in timing from exposure to follow-up.

| | |
|---|---|
| **Reviewer** | **3** |
| **Name** | **Crawford, Joel** |
| **Affiliation** | **Linkoping University Department of Health Medicine and Caring Sciences** |
| **Date** | **17-Sep-2025** |
| **COI** | **None** |

Overall, this is a very interesting protocol and is timely as newer research is showing even low levels of alcohol consumption increase cancer risk. However, I have raised some issues noted below:

Introduction

- The authors need to further develop the argument for the need of updating label messages. Currently they mention existing labels have not been evaluated or are poorly designed, this needs to be elaborated on further. For example, qualitative findings from the UK highlights issues with current industry led responsibility messages - individuals typically perceive that labels are not salient to them as they position themselves as unproblematic drinkers, whilst messages reflecting personal responsibility were perceived as an industry ploy. However, they also suggest that health messages regarding cancer risk are potentially impactful. These findings highlight the need for updating label messages and adds credence to the study's focus on cancer warnings - https://doi.org/10.1111/bjhp.12627

- The introduction needs more justification for the study manipulation of label positioning. Currently there is a brief mention in the objectives subsection, but this needs to come earlier in the introduction – it could be linked to the note on the Yukon study results being mediated by consumers' attention and cognitive processing of the labels.

- In addition to this, the study has referenced positioning of nutritional labelling, however it would add credence to discuss health risk behaviours – health risk and health promotion information is processed differently. There is evidence regarding this topic for tobacco and whilst not directly testing positioning, evidence highlights consumers allocate little viewing time generally to alcohol warnings unless labels are made salient and placed prominently, i.e., front and upper panel (see https://doi.org/10.1016/j.puhe.2024.07.030)

- Extant literature has been noted as having short follow-up periods, however the proposed study also has a short 1-month follow-up period. Can this follow-up period really be enough to address this gap in the literature - especially as other online studies such as Brennan et al., (2022) have also used the same follow-up period? Justification is needed.

- The introduction would benefit from a discussion on why or how the experimental cancer warning labels will impact the secondary outcomes – such detailing is apt for a study protocol. There is mention of behaviour change models, however this needs to be elaborated on.

Methods and Analysis

- The plan for accounting for SES in the setting/recruitment subsection is great, well done.

- Whilst the study proposes an ecologically valid recruitment method, have the authors any plan for accounting for potential sampling biases? For example, recruiting individuals at point of sale is likely incur social desirability bias, individuals may feel less comfortable to disclosing heavy or risky drinking patterns, especially if they are with others. Additionally, it should be noted or considered that point of sale recruitment may lead to other issues such as time pressures, increased cognitive load or distractions (especially at check outs), these factors may also impact baseline survey responses.

- Further to this and importantly, assessing knowledge of risk be underestimated at point of sale as individuals potentially may wish to supress or downplay risks right after the purchase of alcohol compared to other times or mediums (i.e., it may result in higher defensive processing). This can potentially inflate any differences found between baseline scores and the follow-up; hence the potential for 'mode effects' should be made transparent.

- Will participants be informed of the reimbursement prior to or after consent? This could potentially impact selection bias.

- Can the authors be certain that the simple randomisation procedure will result in an equal allocation between intervention and control groups? This needs to be justified in lieu of using other procedures such as block randomisation.

- Typo on line 21 of page 11, 'designed' has been written twice.

- What is the justification for the threshold of 1.2% ABV? This should be discussed.

- It’s good that the authors have accounted for length of exposure to the labels, but again social desirability may influence these responses, this should be highlighted.

- Risk perceptions typically assess threats to health in the dimensions of perceived vulnerability and perceived susceptibility. The proposed single item only assesses susceptibility. The authors should consider an additional item that assesses vulnerability or provide justification for targeting only susceptibility.

- Can the authors justify a single item for assessing intentions, this construct is typically assessed using 2-4 items in the literature, that usually consider both plans and intentions within a specific time frame.

- Emotional responses to the labels will only be measured among those reporting to have noticed the labels – earlier in the manuscript it’s noted that emotional responses to the labels will be assessed with the survey items. Please clarify this.

- Again, the notion of only assessing those who have noticed the labels will be applied to the product appeal item. Does this mean if participants say nothing about the labels you won’t ask them to complete the items?

- The use of fear appeals in health communication has produced mixed results, however for individuals that engage with heavy or risky drinking the use of such appeals often results in defensiveness; hence the messages may have limited impact on this group. Is there a plan to account for this factor in the analyses, not sure if I missed this but I can only see demographics being mentioned?

- Is there a plan to assess any data collected in the open-ended section of the survey? This could provide valuable insights into potential acceptability and uptake of the intervention.

Discussion/Limitations

- There is mention that the study will provide evidence if the knowledge increase will be sustained over a longer term – however the plan is to follow-up only 1-month later. This should be revised or rephrased.

- The authors cannot be certain that participants will simply not remove the labels afterwards – this should be considered.

This protocol is very interesting and has potential real-world applications for policy and practice. Good luck with the revisions and I look forward to seeing the publication of the results.

---

| | |
|---|---|
| **Reviewer** | **4** |
| **Name** | **Dack, Kyle** |
| **Affiliation** | **University of Bristol, MRC Integrative Epidemiology Unit** |
| **Date** | **10-Oct-2025** |
| **COI** | **None** |

---

Statistical review summary:

The protocol clearly pre-specifies the procedures which will be followed during this RCT. For the most part it is comprehensive and aligned with the relevant reporting checklist. My primary concern is the plan to perform significance testing on baseline characteristics between groups, which is not recommended because we already know any differences occurred by chance. I also suggest adding more information regarding the sample size calculation, with details below.

1. Checklist: The authors have applied the SPIRIT 2013 checklist and the methodology is thoroughly described. However, there is a more up-to-date version of this checklist – SPIRIT 2025, which I recommend applying. This is relevant to some of the comments below.

Abstract:

2. The information in the abstract is clear and for the most part aligns with the SPIRIT guidelines. However, I suggest adding the key inclusion or exclusion criteria, and sample size goal. I also suggest reorganizing to align with the sections suggested in the SPIRIT elaboration document, which may enhance the readability: https://pmc.ncbi.nlm.nih.gov/articles/PMC12128891/ [item 1b]

3. Study design: After reading the study objectives and hypotheses sections, the impression given was that this will employ a 2x2 design. However, in “study design” it is explained that it is 2x2x2, with the third effect being “iii) pre-test for the primary outcome (present or absent).” This aspect of the design was not mentioned in prior sections as far as I can see, and it seems to be the first mention of there being a pre-test. Because of this, it is not clear exactly what is being tested or why, and it may be clearer if it could be integrated briefly into the prior sections.

4. I suggest the authors consider the use of a participant timeline, which is part of SPIRIT 2025 and is a nice way of summarising the data collection items and timing. An example is available here:: https://www.bmj.com/content/389/bmj-2024-081660 and the template is available here: https://www.consort-spirit.org/

5. Sample size estimation – primary outcome. There are some important elements missing from the primary outcome estimation – which software/formula was used? What was the desired power and alpha? Given that this is a 2x2x2 design, was this based on all of these factors or a single effect? How was the primary outcome sample size calculated? It would also improve the clarity to briefly report the endpoint, timepoint, and how the 125 breaks down per-cell. If this is the total for the 2x2x2 there are 8 cells which does not cleanly divide 125 participants.

6. Sample size estimation – secondary outcome. Similar to the above could the authors please clarify how the factorial design fits with this calculation.

7. "To assess whether randomization was successfully applied, baseline characteristics will be compared across experimental conditions. Statistically significant differences in sociodemographic variables (age, gender, education level), alcohol consumption, and prior knowledge of alcohol-related harms will be examined. Chi-square tests will be used for categorical variables, while one-way ANOVA tests will be used for continuous variables." While a descriptive comparison of baseline characteristics is reasonable, it is not recommended to perform significance testing on randomized groups because this lacks meaning – we already know that any differences are due to chance and the null hypothesis must be correct: https://www.nature.com/articles/s41393-018-0203-y. https://tidsskriftet.no/en/2020/03/medisin-og-tall/avoid-significance-tests-background-variables-randomised-controlled-trials, https://www.sciencedirect.com/science/article/pii/S0959804923006597

8. There is mention of "Bonferroni corrections for multiple comparisons where necessary." But this should be possibly to determine in advance and report in the protocol.

9. The analysis states that linear regression will be used for continuous variables. I suggest the authors plan to assess the distribution first, and consider Poisson for count data if it is skewed, or negative-binomial if there is over-dispersion.

---

## VERSION 1 - AUTHOR RESPONSE

Reviewer: 1
Dr. Yury Evgeny Razvodovsky, Grodno State Medical University, Grodno State Medical University
Comments to the Author:
The aims of this research project is to evaluate the longer-term impacts of a cancer warning label on alcohol containers relative to a control label on behavioral outcomes. This is well-designed study that addressed an important alcohol policy issue, which remains surprisingly underdeveloped. Methodologically there appear to be no critical weaknesses. I have no any specific comments.

**Response**: Thank you very much for the feedback and taking the time to review the study.

Reviewer: 2
Prof. Colin P. Green , Norwegian University of Science and Technology

Comments to the Author:

"Impacts of Alcohol Health Warning Labels in a Real-World Setting: Protocol for a Randomised Controlled Trial Among Supermarket Customers in Barcelona"

This protocol sets out a research plan to examine the effect of exposure to health warning labelling on alcohol on knowledge of health risks and health behaviors. It does so using a randomized design where cancer warning labels or standard health information labels are applied to alcohol containers of individuals who have recently purchased alcohol.

In general, the protocol is clear, well-described, and the research design carefully considered. I have some comments that I feel should be further considered.

**Response**: Thank you very much for your feedback and constructive comments. We have responded to your comments below.

Comments:

1. The main set of concerns relate to statistical power:

a.The treatment is the application of the label to one alcoholic container. These can range (if I understand it correctly) from one beer, a bottle of wine, or bottle of spirits. In the case of the former two, this is likely a very short-term form of exposure. I.e. In many cases these will be consumed relatively quickly and disposed of. Would we expect then this exposure to this information to plausibly have large medium-term (2 months) effects. For instance, on page 13 treatment effects of 1.5 to 2 standard drinks a week. These seems large.

**Response**: The differences in exposure inherent in the design are indeed limitation to the study (as noted in the limitations section) – and it's one among the reasons we focus on knowledge as the main outcome, as it's more likely to have longer term effects also after the short exposure. Our previous research (Correia et al 2024) showed very high effect sizes after brief exposure, and in this research, we are interested how mid-to-longer term exposure to the information influences those effects.

The secondary outcomes such as behaviour are added in order to gather a wider range of data from such a study, the sample size calculations are based on available data from previous studies, but also on the sample size reachable based on the available budget. Considering the limitations in exposure differences and its possible impact on longer term effects, the study thus includes not only behaviour, but also behavioural precursors, as well as alternative roles of alcohol labels, as those are more likely to be affected already by shorter exposure.

b. Related to this, I guess in some cases the alcohol is not (solely) drunk by the purchaser. As we don't

know actual exposure to the message this gives the estimates an additional ITT interpretation and has implications for statistical power.

**Response**: To address this, we are asking the participants whether the alcohol they have purchased is for their own consumption to ensure we are not labelling alcohol that will be given to other people, as well as how they are consuming the alcohol (on their own, with others etc). given the current context of the study, exposure by more than one person to the message (for example by bottle of wine being on the table while people are sharing it) does not take away from the individual effects.

c. Response rate of 50% at second follow up. What is this based on? Is this conservative?

**Response**: The 50% response rate is based on the estimations of the market research company and is on the conservative side. We have added this information to the sample size calculation section.

d. The study aims to test several hypotheses. This is discussed briefly in one sentence (multiple comparisons), but should this be part of the calculation of statistical power?

**Response**: The different hypotheses are not overlapping; thus we did not expect this to have any effect on the statistical power. As this is a RCT, the sample size has to assure that the main hypothesis with the primary outcome is well powered, which was our focus. The secondary outcomes are also considered however, and we have done that by increasing the sample size in line with budgetary possibilities and calculating what differences in behavioural variables would that detect.

e. In general, my concern is that the study as described now may end in a set of uninformative null results.

**Response**: We are aiming to replicate our previous online experiment in which the knowledge effects were very high, for this reason our hypothesis is that cancer message will increase the associated knowledge, even if at smaller magnitude.

2. Related to this are questions of external validity.

a. The underlying policy question is whether labelling changes behavior. The real-world setting would be moving to a setting where all alcohol was labelled. Here we examine the effect of labelling one drink container. I agree this is more realistic than lab-based approaches but has issues re: statistical power above.

**Response**: As described in the introduction, labelling's focus is not behaviour change but has multiple possible roles, which we are also evaluating in this study (increasing knowledge, increasing policy support, decreasing product appeal). We agree the design has limitations, but we have attempted to do the most optimal design given the constraints (in order for all the products to be labelled we would need either legislation or organisational buy-in, and so far only one study (Yukon study) was able to guarantee that, while all the others were done online. As mentioned above, the statistical power for the primary outcome was based on our previous online experiment (Correia et al 2024).

b. A second related issue is that the act of applying the label is, as discussed, naturally known to subjects and one might be concerned about interviewer demand effects (i.e. that subjects who realize they have

been subject to a health treatment respond in a specific way in follow-ups). It is stated that these types of effects are differenced out by the study design (all receive a label). It is not entirely clear that this is true. One related question – what is the status quo – is alcohol regularly labelled with something like the control label?

**Response**: We agree with this issue, and we have revised the limitations section to adress this more directly– it is true that there might be an effect of label being applied in front of participant that goes beyond the design, and that the act of the research itself might have an influence on the results.

At this point, the topics presented occasionally on the control label appear on existing alcohol labels (predominantly pregnancy, also drink driving and underage, responsible drinking messages based on our unpublished data), however they do not appear with the existing visual elements (large, colorful, clearly separated from the rest of the label). The existing labels are thus less visible.

c. Finally, a concern in the real-world setting is that initial effects of the policy may 'wash-out' over time as people get used to the warnings. Here this type of effect (which is discussed) is different in that it represents differences in timing from exposure to follow-up.

**Response:** We address this issue in the revised Limitations section of the Discussion.

Reviewer: 3
Dr. Joel Crawford, Linkoping University Department of Health Medicine and Caring Sciences
Comments to the Author:

Overall very interesting and I am eager to see how the RCT goes.

**Response**: Thank you for the careful consideration of the manuscript and for the helpful comments. Please see our responses below.

Overall, this is a very interesting protocol and is timely as newer research is showing even low levels of alcohol consumption increase cancer risk. However, I have raised some issues noted below:

Introduction
• The authors need to further develop the argument for the need of updating label messages. Currently they mention existing labels have not been evaluated or are poorly designed, this needs to be elaborated on further. For example, qualitative findings from the UK highlights issues with current industry led responsibility messages - individuals typically perceive that labels are not salient to them as they position themselves as unproblematic drinkers, whilst messages reflecting personal responsibility were perceived as an industry ploy. However, they also suggest that health messages regarding cancer risk are potentially impactful. These findings highlight the need for updating label messages and adds credence to the study's focus on cancer warnings - https://doi.org/10.1111/bjhp.12627

**Response**: This has now been elaborated in the introduction through adding additional references and focusing on the limitations of the voluntary warnings next to the mandatory.

• The introduction needs more justification for the study manipulation of label positioning. Currently there is a brief mention in the objectives subsection, but this needs to come earlier in the introduction – it could be linked to the note on the Yukon study results being mediated by consumers' attention and cognitive processing of the labels.

**Response**: This has now been addressed through adding a paragraph in the introduction that focuses on justification for inclusion of label positioning as key variable.

• In addition to this, the study has referenced positioning of nutritional labelling, however it would add credence to discuss health risk behaviours – health risk and health promotion information is processed differently. There is evidence regarding this topic for tobacco and whilst not directly testing positioning, evidence highlights consumers allocate little viewing time generally to alcohol warnings unless labels are made salient and placed prominently, i.e., front and upper panel (see https://doi.org/10.1016/j.puhe.2024.07.030)

**Response**: This issue has been addressed in the abovementioned paragraph that focuses on justification for inclusion of label positioning as key variable.

• Extant literature has been noted as having short follow-up periods, however the proposed study also has a short 1-month follow-up period. Can this followup period really be enough to address this gap in the literature - especially as other online studies such as Brennan et al., (2022) have also used the same follow-up period? Justification is needed.

**Response**: This has been justified by positioning the Brennan et al study as online-only as opposed to the current study that studies the labels on containers in real world.

• The introduction would benefit from a discussion on why or how the experimental cancer warning labels will impact the secondary outcomes – such detailing is apt for a study protocol. There is mention of behaviour change models, however this needs to be elaborated on.

**Response:** The introduction has been adapted to better reflect the existing literature also on the secondary outcomes.

Methods and Analysis

• The plan for accounting for SES in the setting/recruitment subsection is great, well done.

**Response**: Thank you.

• Whilst the study proposes an ecologically valid recruitment method, have the authors any plan for accounting for potential sampling biases? For example,

recruiting individuals at point of sale is likely incur social desirability bias, individuals may feel less comfortable to disclosing heavy or risky drinking patterns, especially if they are with others. Additionally, it should be noted or considered that point of sale recruitment may lead to other issues such as time pressures, increased cognitive load or distractions (especially at check outs), these factors may also impact baseline survey responses.

**Response**: This has not been addressed in the Limitations section of the Discussion – unfortunately there are inherent biases in the absence of the possibility to put labels on all beverages in-store and recruiting the individuals directly. One of the inclusion criteria is purchase of the alcohol beverage rather that the (quantity of) the self-reported consumption, which will decrease the issue of disclosing heavy or risky drinking. Additionally, we will collect data on demographics of (eligible) people who refuse to participate.

• Further to this and importantly, assessing knowledge of risk be underestimated at point of sale as individuals potentially may wish to supress or downplay risks right after the purchase of alcohol compared to other times or mediums (i.e., it may result in higher defensive processing). This can potentially inflate any differences found between baseline scores and the follow-up; hence the potential for 'mode effects' should be made transparent.

**Response**: Thank you for pointing this out. We have included that in the Limitations section.

• Will participants be informed of the reimbursement prior to or after consent? This could potentially impact selection bias.

**Response**: All participants will be informed of reimbursement prior to having to provide informed consent, in order to have all the information necessary to make a decision. This has been now clarified in the methods section of the manuscript and reflected on to the Limitations section.

• Can the authors be certain that the simple randomisation procedure will result in an equal allocation between intervention and control groups? This needs to be justified in lieu of using other procedures such as block randomisation.

**Response**: While simple randomization does not guarantee exact equal allocation at any given sample size, with our target sample of 1300 participants, the probability of substantial imbalance is low. Statistical simulations demonstrate that with samples over 100 participants, simple randomization typically produces group sizes within 5-10% of perfect balance. Any minor imbalances will be addressed analytically through covariate adjustment in our regression models. We chose simple randomization over block randomization to maintain allocation unpredictability and avoid potential selection bias that could occur if research staff become aware of the blocking structure during recruitment.

• Typo on line 21 of page 11, 'designed' has been written twice.

**Response**: Thank you for noticing, this has been fixed.

• What is the justification for the threshold of 1.2% ABV? This should be discussed.

**Response**: This is the official legal definition of alcoholic beverage both in EU and Spain. This has now been clarified in the manuscript.

• It's good that the authors have accounted for length of exposure to the labels, but again social desirability may influence these responses, this should be highlighted.

**Response**: Thank you for noting, this is now highlighted in the Limitations section that has been significantly reworked.

• Risk perceptions typically assess threats to health in the dimensions of perceived vulnerability and perceived susceptibility. The proposed single item only assesses susceptibility. The authors should consider an additional item that assesses vulnerability or provide justification for targeting only susceptibility.

**Response:** In this study, we are using the same items as in our previous online study to be able to compare the results more directly. We agree with the comment however, and we will take the comment on board for the future studies.

• Can the authors justify a single item for assessing intentions, this construct is typically assessed using 2-4 items in the literature, that usually consider both plans and intentions within a specific time frame.

Response: Same as above, in this study, we are using the same items as in our previous online study to be able to compare the results more directly. Given that we wanted to keep the survey succinct and that we also have behavioural variables, we only chose one variable to measure the intentions.

• Emotional responses to the labels will only be measured among those reporting to have noticed the labels – earlier in the manuscript it's noted that emotional responses to the labels will be assessed with the survey items. Please clarify this.

**Response**: The emotional response will be measured in the survey, but only for the subset of participants who have reported noticing the labels, as the questions directly refer to how being exposed to the label made them feel (if this question would be posed also to the ones not noticing the labels, their responses would be meaningless as they did not notice the labels).

• Again, the notion of only assessing those who have noticed the labels will be applied to the product appeal item. Does this mean if participants say nothing about the labels you won't ask them to complete the items?

**Response**: Indeed, assessing the product appeal as operationalized through items in our study is relevant only for the participants that have noticed the labels. This will be asked in a separate question that will determine whether the participants will be asked the questions on product appeal.

• The use of fear appeals in health communication has produced mixed results, however for individuals that engage with heavy or risky drinking the use of such appeals often results in defensiveness; hence the messages may have limited impact on this group. Is there a plan to account for this factor in the analyses, not sure if I missed this but I can only see demographics being mentioned?

**Response**: Yes, this will be accounted for by dividing them in low and high risk drinkers based on their AUDIT score. This has now been clarified in the data analysis plan.

• Is there a plan to assess any data collected in the open-ended section of the survey? This could provide valuable insights into potential acceptability and uptake of the intervention.

**Response**: Yes, while this is not the primary purpose of the study, we will look into that data through thematic analysis to complement the main results. This has been added to the data analysis section.

Discussion/Limitations

• There is mention that the study will provide evidence if the knowledge increase will be sustained over a longer term – however the plan is to follow-up only 1-month later. This should be revised or rephrased.

**Response**: Given the current state of alcohol labelling literature, 1-month is already longer term (as opposed to pre-post online experiments that are mainly being conducted) – hence the choice of this term.

• The authors cannot be certain that participants will simply not remove the labels afterwards – this should be considered.

**Response**: We have considered this when developing the questionnaire, and a question on whether the purchased containers still contain the stickers is included as part of the follow-up on questionnaires.

This protocol is very interesting and has potential real-world applications for policy and practice. Good luck with the revisions and I look forward to seeing the publication of the results.
Joel Crawford PhD,
Linköping Universitetet

**Response:** Thank you very much for your time and feedback.

Reviewer: 4
Dr. Kyle Dack, University of Bristol
Comments to the Author:

Statistical review summary:

The protocol clearly pre-specifies the procedures which will be followed during this RCT. For the most part it is comprehensive and aligned with the relevant reporting checklist. My primary concern is the plan to perform significance testing on baseline characteristics between groups, which is not recommended because we already know any differences occurred by chance. I also suggest adding more information regarding the sample size calculation, with details below.

**Response**: Thank you very much taking the time to review the manuscript and providing comments, they are responded to below.

1. Checklist: The authors have applied the SPIRIT 2013 checklist and the methodology is thoroughly described. However, there is a more up-to-date version of this checklist – SPIRIT 2025, which I recommend applying. This is relevant to some of the comments below.

**Response**: We have revised the protocol accordingly with the new SPIRIT checklist. Just for clarification, as the paper was being prepared, the new checklist was not published yet, hence we used the available one from 2013.

Abstract:
2. The information in the abstract is clear and for the most part aligns with the SPIRIT guidelines. However, I suggest adding the key inclusion or exclusion criteria, and sample size goal. I also suggest reorganizing to align with the sections suggested in the SPIRIT elaboration document, which may enhance the readability: https://pmc.ncbi.nlm.nih.gov/articles/PMC12128891/ [item 1b]

**Response**: We have added the inclusion and exclusion criteria and sample size goal in the abstract. The abstract format remained as requested by the journal formatting guidelines. The trial has however been registered in clinicaltrials.gov and all relevant information is provided in on the website.

3. Study design: After reading the study objectives and hypotheses sections, the impression given was that this will employ a 2x2 design. However, in "study design" it is explained that it is 2x2x2, with the third effect being "iii) pre-test for the primary outcome (present or absent)." This aspect of the design was not mentioned in prior sections as far as I can see, and it seems to be the first mention of there being a pre-test. Because of this, it is not clear exactly what is being tested or why, and it may be clearer if it could be integrated briefly into the prior sections.

**Response**: Thank you for noting this, this has now been integrated in the introduction rather than only mentioned in the methods.

4. I suggest the authors consider the use of a participant timeline, which is part of SPIRIT 2025 and is a nice way of summarising the data collection items and timing. An example is available here: https://www.bmj.com/content/389/bmj-2024-081660 and the template is available here: https://www.consort-spirit.org/

**Response**: The participant timeline has been added in Appendix.

1. Sample size estimation – primary outcome. There are some important elements missing from the primary outcome estimation – which software/formula was used? What was the desired power and

alpha? Given that this is a 2x2x2 design, was this based on all of these factors or a single effect? How was the primary outcome sample size calculated? It would also improve the clarity to briefly report the endpoint, timepoint, and how the 125 breaks down per-cell. If this is the total for the 2x2x2 there are 8 cells which does not cleanly divide 125 participants.

**Response** We thank the reviewer for this helpful comment and have expanded the description of the primary outcome sample size calculation accordingly. The calculation now specifies that it was based on a two-sided comparison of proportions with a significance level of 0.05 and 80% power, conducted in R using the power.prop.test() function. Using a conservative baseline prevalence of 13% and a target odds ratio of 2.5, the required sample size was 125 participants per message type group to test the main effect of message type, rather than the full 2×2×2 factorial design. The corresponding total sample size is therefore N = 250. This clarification has been added to the manuscript.

6. Sample size estimation – secondary outcome. Similar to the above could the authors please clarify how the factorial design fits with this calculation.

**Response**: The behavioural analyses will incorporate the factorial design structure within a multiple regression framework. The sample size for the secondary outcome (N = 650) was estimated using an F-test for a block of five predictors under an unconditional model, with $\alpha = 0.05$, power = 0.80, and $f^2 = 0.02$. The manuscript has been revised to include this clarification.

7. "To assess whether randomization was successfully applied, baseline characteristics will be compared across experimental conditions. Statistically significant differences in sociodemographic variables (age, gender, education level), alcohol consumption, and prior knowledge of alcohol-related harms will be examined. Chi-square tests will be used for categorical variables, while one-way ANOVA tests will be used for continuous variables." While a descriptive comparison of baseline characteristics is reasonable, it is not recommended to perform significance testing on randomized groups because this lacks meaning – we already know that any differences are due to chance and the null hypothesis must be correct: https://www.nature.com/articles/s41393-018-0203-y. https://tidsskriftet.no/en/2020/03/medisin-og-tall/avoid-significance-tests-background-variables-randomised-controlled-trials, https://www.sciencedirect.com/science/article/pii/S0959804923006597

**Response**: We have read the suggested references, and - as we do not have an suspicion that randomization procedures did not work - we will not be testing baseline characteristics but only provide descriptives. This has been changed in the revised protocol.

8. There is mention of "Bonferroni corrections for multiple comparisons where necessary." But this should be possibly to determine in advance and report in the protocol.

**Response**: We thank the reviewer for this helpful observation. The reference to Bonferroni corrections was originally included to remain on the conservative side regarding potential multiple comparisons. After considering the reviewer's comment, we agree that the analysis plan described in this manuscript does not require adjustments for multiple comparisons, as the specified outcomes and hypotheses are predefined and independent analyses are not being performed within the same family of outcomes. We have therefore removed the mention of Bonferroni correction from the manuscript.

9. The analysis states that linear regression will be used for continuous variables. I suggest the authors

plan to assess the distribution first, and consider Poisson for count data if it is skewed, or negative-binomial if there is over-dispersion.

**Response**: We appreciate this useful suggestion and have revised the analysis section accordingly. The updated text now specifies that secondary outcomes measured on five-point Likert scales will be analysed using linear regression, while the outcome measuring the number of standard drinks will first be assessed for its distribution and analysed using linear regression or Poisson regression, as appropriate. In the presence of overdispersion, a negative binomial regression will be applied. This revision ensures that model selection reflects the empirical distribution of the data.

---

## VERSION 2 - REVIEW

---

| | |
|---|---|
| **Reviewer** | **2** |
| **Name** | **Green , Colin P.** |
| **Affiliation** | **Norwegian University of Science and Technology** |
| **Date** | **12-Dec-2025** |
| **COI** | |

---

Thank you for your responses. In particular the revised protocol is much clearer on potential limitations. I remain of the view that this is an exciting research project.

---

| | |
|---|---|
| **Reviewer** | **4** |
| **Name** | **Dack, Kyle** |
| **Affiliation** | **University of Bristol, MRC Integrative Epidemiology Unit** |
| **Date** | **27-Nov-2025** |
| **COI** | |

---

Thank you to the authors for their constructive response to my queries/comments, it will be interesting to see the results of the study.
